# Non-Invasive Brain-Computer Interfaces: Converging Frontiers in Neural Signal Decoding and Flexible Bioelectronics Integration

**DOI:** 10.1007/s40820-025-02042-2

**Published:** 2026-01-12

**Authors:** Sheng Wang, Xiaobin Song, Xiaopan Song, Yang Gu, Zhuangzhuang Cong, Yi Shen, Linwei Yu

**Affiliations:** 1 College of Electronic and Optical Engineering & College of Flexible Electronics (Future Technology), Nanjing University of Posts and Telecommunications, Nanjing, 210023 People’s Republic of China; 2 Department of Cardiothoracic Surgery, Jinling Clinical Medical College, Nanjing University of Chinese Medicine, Nanjing, 210007 People’s Republic of China; 3 School of Electronics Science and Engineering, Nanjing University, Nanjing, 210023 People’s Republic of China; 4 Department of Cardiothoracic Surgery, Jinling Hospital, Affiliated Hospital of Medical School, Nanjing University, Nanjing, 210007 People’s Republic of China

**Keywords:** Non-invasive BCIs, Deep learning, Neural signal decoding, Nanowires, Flexible bioelectronics

## Abstract

The development of non-invasive brain-computer interfaces (BCIs) relies on multidisciplinary integration across neuroscience, artificial intelligence, flexible electronics, and systems engineering. Recent advances in deep learning have significantly improved the accuracy and robustness of neural signal decoding. Parallel progress in electrode design—particularly through the use of flexible and stretchable materials like nanostructured conductors and novel fabrication strategies—has enhanced wearability and operational stability. Nevertheless, key challenges persist, including individual variability, biocompatibility limitations, and susceptibility to interference in complex environments. Further validation and optimization are needed to address gaps in generalization capability, long-term reliability, and real-world operational robustness. This review systematically examines the representative progress in neural decoding algorithms and flexible bioelectronic platforms over the past decade, highlighting key design principles, material innovations, and integration strategies that are poised to advance non-invasive BCI capabilities. It also discusses the importance of multimodal data fusion, hardware-software co-optimization, and closed-loop control strategies. Furthermore, the review discusses the application potential and associated engineering challenges of this technology in clinical rehabilitation and industrial translation, aiming to provide a reference for advancing non-invasive BCIs toward practical and scalable deployment.

## Highlights

- The latest advancements in neural signal decoding and the integration of flexible bioelectronics for non-invasive brain-computer interfaces are reviewed.
- Multimodal data fusion, hardware-software co-optimization, and closed-loop control strategies are critical for enhancing the robustness, adaptability, and real-time performance of brain-computer interface (BCI) systems.
- The robust real-world deployment of BCIs requires breakthroughs in cross-subject generalization, environmental adaptability, and system reproducibility.

## Graphical abstract

**Figure:**
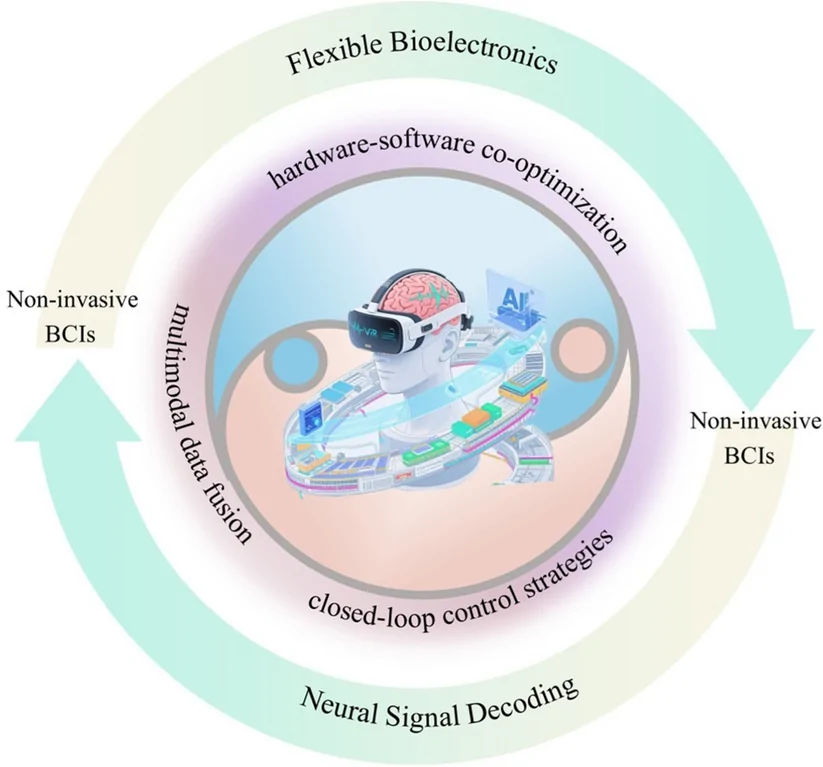

## Introduction

The development of brain-computer interfaces (BCIs) represents a significant integration of neuroscience, artificial intelligence (AI), and bioelectronics, establishing direct communication pathways between neural activity and external technological systems [1–6]. This integration enables effective control of computer interfaces, assistive devices, and robotic platforms, thereby enhancing human-technology interactions [7–11]. Specifically, BCIs are broadly categorized into two types: invasive and non-invasive. Invasive BCIs, which utilize direct electrode-neural tissue contact, typically achieve high signal-to-noise ratio (SNR), sensitivity, and resolution, making them suitable for precision interventions requiring high signal quality. However, their application is constrained by surgical risks, biocompatibility issues, and long-term stability challenges. At present, invasive BCIs are mainly used in clinical settings for advanced cognitive research and motor function restoration in paralyzed patients, which may limit near-term direct translation to broader populations, including healthy individuals [12–21]. In contrast, non-invasive BCIs, which do not require surgery and offer high safety, have demonstrated broad scalability and applicability in clinical practice. These systems are applied in various domains, such as communication, rehabilitation, virtual reality (VR) and augmented reality (AR), cognitive impairment, mental health, fatigue and vigilance monitoring, chronic inflammation, and autonomic nerve regulation [22–24]. Clinically, they serve as valuable non-pharmacological interventions for conditions like amyotrophic lateral sclerosis (ALS) and stroke rehabilitation, contributing to improvements in patients' quality of life [5, 25, 26]. For instance, Sellers et al. [27] proposed a non-invasive BCI system that restored essential communicative functions in ALS patients, enabling reliable letter selection and spontaneous communication in over two-thirds of trials. Similarly, Biasiucci et al. [26] integrated a non-invasive BCI with functional electrical stimulation, showing improvements among chronic stroke patients. Beyond its medical applications, BCI technology is being increasingly applied in entertainment and automotive safety domains, highlighting its broad societal impact [28–31].

Electroencephalography (EEG) remains the primary signal acquisition method for non-invasive BCIs, valued for its non-invasiveness, high temporal resolution, and clinical applicability [32, 33]. However, EEG signals are susceptible to physiological artifacts and environmental electromagnetic interference, which result in a low SNR in real-world environments, thereby lowering signal quality and affecting the reliability of subsequent analysis [34]. Other non-invasive neuroimaging technologies face similar challenges. For instance, functional near-infrared spectroscopy (fNIRS) and functional magnetic resonance imaging (fMRI) are constrained by low temporal resolution. In contrast, magnetoencephalography (MEG), despite its higher spatiotemporal resolution, requires strict environmental conditions [35–38]. Furthermore, conventional decoding paradigms, including motor imagery (MI), steady-state visual evoked potentials (SSVEP), and P300 event-related potentials (ERP), are constrained by factors such as dependence on user state and individual variability. These limitations impede their ability to achieve high robustness in real-world scenarios [39–42]. Therefore, the development of non-invasive neural recording technologies with millisecond-scale temporal resolution and high SNR remains a key direction for advancing the practical application of high-performance BCIs [43].

To support more reliable performance in real-world applications, recent research is increasingly focused on two directions: advancing neural signal decoding through AI and integrating flexible bioelectronic platforms. The rapid progress of deep learning architectures [44–46], especially convolutional neural networks (CNNs), multimodal hybrid networks, and Transformer models, has improved decoding accuracy and system operational stability, with performance surpassing that of traditional machine learning methods [47–50]. Meanwhile, multimodal technological strategies are continuously expanding the boundaries of non-invasive neural monitoring, offering new insights for analyzing spatiotemporal neural dynamics and their regulatory mechanisms [51]. For instance, multimodal stimulation paradigms that integrate visual and auditory cues have been shown to improve the accuracy and robustness of neural signal decoding. In summary, these technological trends indicate feasible pathways toward high-performance non-invasive BCIs, although related engineering implementation and clinical translation challenges still need to be addressed [52].

Traditional rigid electrodes, due to their high mechanical stiffness and limited ability to conform to the skin surface, frequently cause discomfort and unstable contact. This can lead to a degradation in neural signal quality over time, potentially affecting the accuracy and reliability of BCIs [53–55]. Recent advances in flexible bioelectronics provide promising solutions for enhancing the electrode-skin interface [56–63]. By employing flexible polymer substrates and stretchable conductive materials, electrodes have shown notable improvements in mechanical compliance, conductivity, and resistance to deformation [53, 64–66], thereby enhancing wearing comfort and signal stability [62, 67, 68]. In particular, electrode designs based on flexible conductive films, nanowire materials, or hydrogels hold promise for reducing electrode-skin interface impedance, mitigating motion artifacts, and improving the SNR [69–77]. Looking ahead, combining these flexible sensing platforms with advanced deep learning decoding methods is expected to further improve the performance of non-invasive BCIs further. Specifically, the innovation in flexible electrodes establishes the hardware foundation for high-performance BCIs. By improving interfacial contact and suppressing motion artifacts, these electrodes provide more stable, high-SNR raw signals, thereby supplying high-fidelity data for subsequent decoding. Building on this platform, advanced algorithms, such as deep learning, leverage their powerful feature learning capabilities not only to achieve significant improvements in decoding performance compared to traditional methods but also to actively compensate for inherent hardware limitations such as residual noise and cross-subject variability. This synergistic paradigm, which embodies the principle of hardware laying the foundation and algorithms driving advancement, forms a positive feedback loop that collectively expands the performance boundaries of non-invasive BCIs [78–80].

**Fig. 1:**
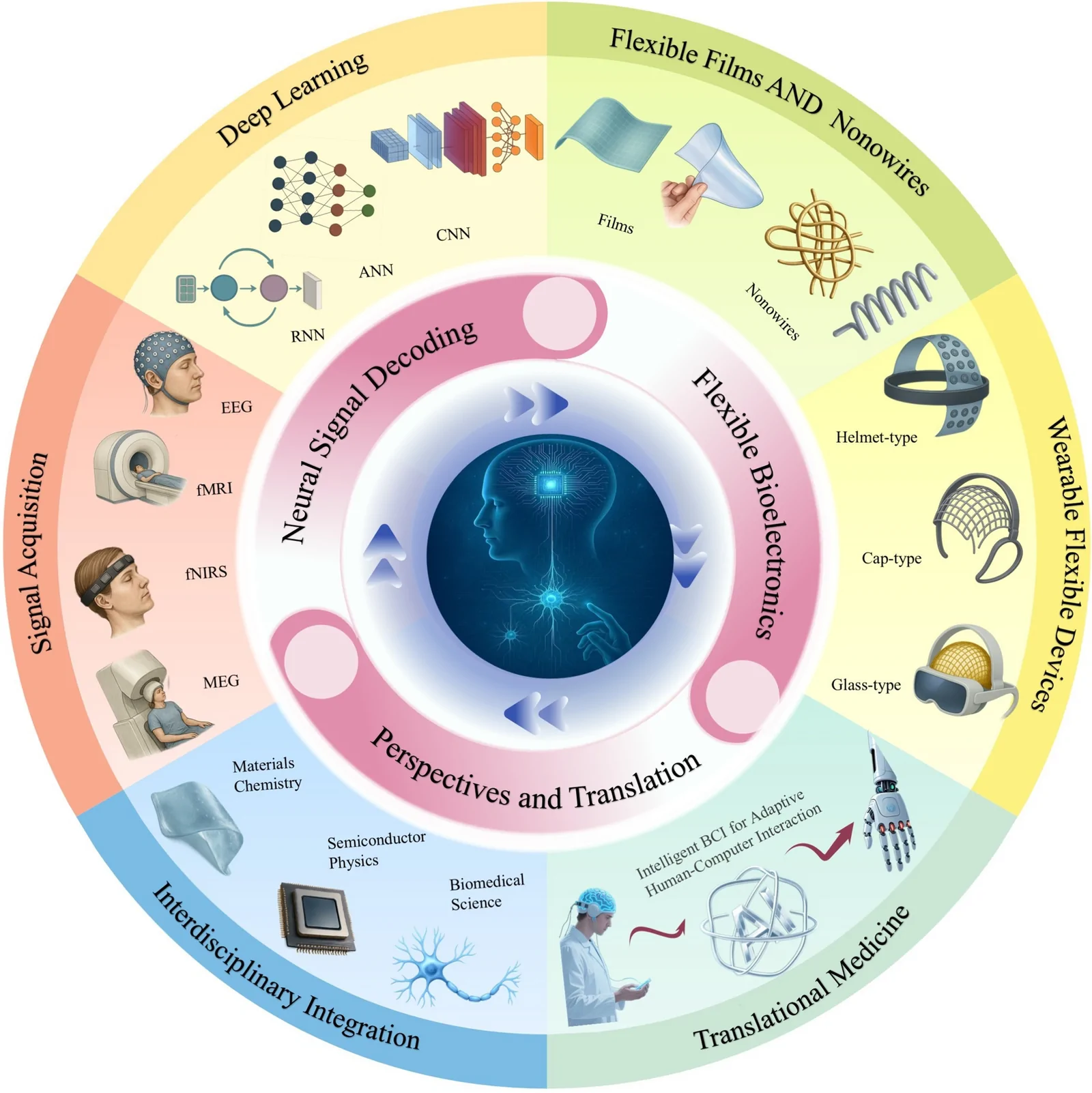
Non-invasive BCI: converging frontiers in neural signal decoding and flexible bioelectronics integration

This review provides a comprehensive overview of the latest advancements in neural signal decoding, flexible bioelectronics, and their synergistic integration in the field of non-invasive BCIs, highlighting their impact on clinical and industrial applications. The article is structured as follows: Following the introduction in Sects. 1 and 2 reviews pivotal advances in neural signal decoding, including multimodal stimulation paradigms, multimodal neural signal acquisition technologies, and the significance of dynamic neural decoding and closed-loop control strategies driven by deep learning. Section 3 critically explores technological innovations in flexible bioelectronics, including interface optimization mechanisms for conductive thin films, miniaturization strategies for nanowire-based devices, multiphysics coupled design of wearable systems, and hardware-software co-optimization with deep learning architectures. Section 4 addresses current technological challenges and future opportunities, focusing on multimodal neural sensing, the enhancement of robustness in adaptive closed-loop systems, and the development of scalable clinical translation pathways. Through a comprehensive analysis of interdisciplinary technological convergence, this review establishes a tripartite framework (decoding-sensing-application) for next-generation non-invasive BCIs and provides actionable insights to support their integration into precision medicine and intelligent human-machine interaction scenarios (Fig. 1).

## Advancing Neural Signal Decoding Methodologies

The core performance limitations of non-invasive BCIs primarily originate from inherent biophysical constraints. When cortical neural activity passes through multiple tissue layers (e.g., the skull and cerebrospinal fluid), it may cause spatial blurring and amplitude attenuation of the signals [81–83]. Consequently, scalp-recorded EEG typically exhibits low spatial resolution, low SNR, and high susceptibility to physiological artifacts and environmental noise. Additionally, individual variations in anatomical structure, physiological states (e.g., fatigue, attention), and psychological states (e.g., motivation, emotion) further exacerbate the non-stationarity and time-varying nature of neural signals [84–86]. These challenges not only undermine the generalization of decoding algorithms but also hinder the stable and reliable decoding of user intent [87, 88].

To address these challenges, a typical neural signal decoding pipeline generally comprises three key stages: preprocessing, feature extraction, and classification. In recent years, with the rapid advancement of deep learning technologies, BCIs have shown improvements in overall performance [89–91]. A notable trend in current research is the shift from "open-loop, static" systems to "closed-loop, adaptive" paradigms [92], with development direction focusing on enhancing real-time decoding capabilities, improving asynchronous detection mechanisms, and optimizing shared control strategies. The core driving force behind this transformation is AI-driven closed-loop human-machine interaction architectures. These architectures not only integrate multimodal sensing and deep learning models but also enable dynamic coordination and bidirectional adaptation between the brain and external devices through a "perceive-decode-applications" closed-loop mechanism [88].

### Multimodal Stimulation Paradigms for Enhanced Neural Decoding

**Fig. 2:**
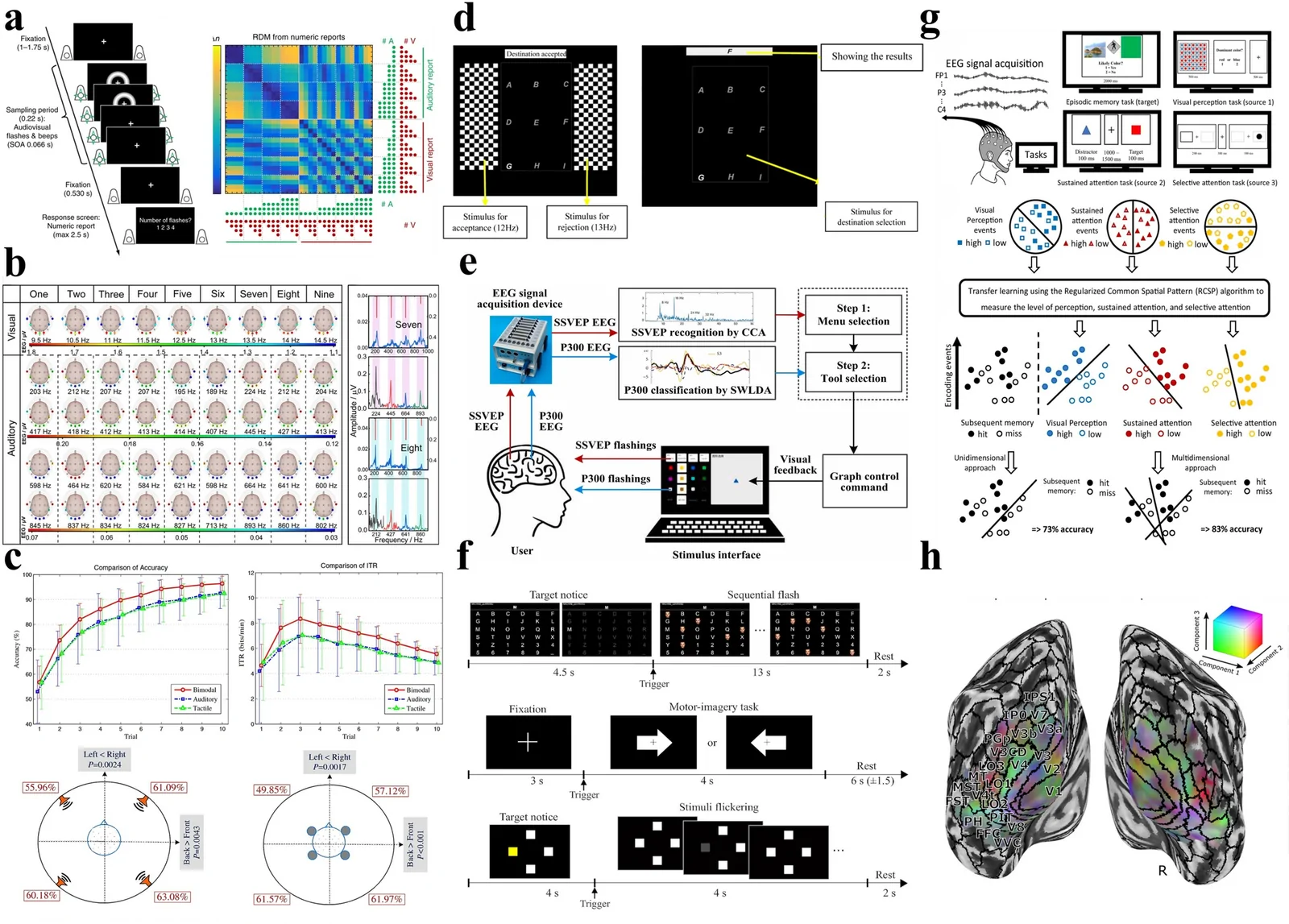
Schematic illustration of multimodal stimulation paradigms for neural signal decoding. **a** Left: An example trial of the flash-beep paradigm. Right: Across-participants' mean illustrating absolute differences. Reproduced with permission [52]. Copyright 2019, Springer Nature. **b** Left: Amplitude distribution of evoked EEG signals in response to the words "one" to "nine". Right: The language and frequency spectra of the feedback EEG signals in MASSR-EEG. Reproduced with permission [98]. Copyright 2023, Wiley-VCH GmbH. **c** System performance under different stimulus conditions and pairwise classification accuracy. Reproduced with permission [99]. Copyright 2016, World Scientific Publishing Company. **d** Stimulation interface for the P300 and SSVEP paradigms. Reproduced with permission [100]. Copyright 2015, IEEE. **e** Framework of the hybrid EEG signal processing module combining SSVEP and P300. Reproduced with permission [41]. Copyright 2022, Elsevier. **f** Experimental designs for the three BCI paradigms. Reproduced with permission [101]. Copyright 2019, Oxford University Press. **g** Multidimensional paradigm combining perception, sustained attention, selective attention, and episodic memory tasks, with transfer learning used to predict memory performance. Reproduced with permission [102]. Copyright 2019, Springer Nature. **h** Visual representation of emotion-related components across cortical visual areas. Reproduced with permission [103]. Copyright 2019, The American Association for the Advancement of Science

Research in non-invasive BCIs is increasingly focusing on the integration of multimodal paradigms to significantly enhance overall system performance. This performance improvement is primarily achieved through advanced multimodal fusion algorithms that effectively integrate information from different sensory channels such as visual, auditory, and tactile, thereby optimizing the accuracy and robustness of neural signal decoding [93, 94]. Specifically, multimodal information processing first requires the construction of efficient fusion algorithms, such as signal analysis based on manifold geometry, feature extraction using common spatial patterns (CSP), and signal calibration leveraging source imaging priors [95–97]. These methods represent preliminary approaches to multimodal integration and modestly improve decoding accuracy. Rohe et al. [52] combined EEG with Bayesian modeling to reveal the neural dynamics of hierarchical Bayesian causal inference in multisensory perception. The work revealed that the brain does not simply integrate sensory signals, but dynamically arbitrates between integration and segregation strategies based on intersensory conflict, computing the final percept through "model averaging". It was further demonstrated that pre-stimulus neural oscillations (such as alpha and gamma power) modulate the causal "prior", thereby elucidating the neural basis of perceptual decision-making with temporal precision. Methodologically, the original authors employed rigorous statistical approaches, including cluster-based multiple comparison correction and bootstrapped confidence intervals, to substantiate their conclusions (Fig. 2a). Notably, Li et al. [98] developed a flexible electrode, which combined SSVEP with multimodal auditory steady-state responses (MASSR). The bimodal paradigm significantly outperformed the unimodal conditions, achieving an average recognition accuracy of 89.6% in the occipital channels (O1, Oz, O2), while the MASSR paradigm attained only 36.7% in the temporal channels (FT9, FT10, TP9, TP10). When the SSVEP-MASSR multimodal paradigm was employed, Li et al. observed that the accuracy improved to 90.4% in the visual channels and 54.0% in the auditory channels. This work served as a proof of concept for the paradigm using the nine words ("one" to "nine"). Extending its applicability to more complex semantic scenarios is a key next step for further development (Fig. 2b). Beyond audiovisual integration, the incorporation of the tactile modality further expands the potential of multimodal BCIs. Yin et al. proposed an innovative auditory-tactile bimodal P300 BCI [99]. The results showed that the bimodal paradigm significantly outperformed the unimodal conditions, achieving an online information transfer rate (ITR) of 10.77 bits min^−1^, which represented an improvement of 45.43% (*p* < 0.05) and 51.05% (*p* < 0.001) over the auditory-only and tactile-only paradigms, respectively. It also achieved a higher average accuracy of 88.67% with fewer trials (average 2.92). Future work exploring its application to more complex scenarios is a promising and anticipated direction for development (Fig. 2c).

Fan et al. [100] developed a hybrid BCI system based on P300 and SSVEP for vehicle destination selection. The system achieved an average accuracy of 98.93% ± 0.48% and a mean selection time of 25.95 ± 1.04 s under real driving conditions. The study represents an important step forward by validating the system's performance in real driving environments with fluctuations in illumination and noise. Future work could systematically evaluate its performance under a broader spectrum of complex and extreme conditions to fully define its operational limits (Fig. 2d).

Tang et al. [41] developed a BCI painting system that employed a hybrid control approach, combining SSVEP and P300. The system achieved an average tool selection accuracy of 88.92% ± 3.94%, with an ITR of 74.20 ± 5.28 bpm in the copy-painting task and 71.80 ± 5.15 bpm in the free-painting task. The average tool selection accuracy was slightly higher than that of the traditional P300-only system (86.78% ± 4.56%), while the ITR was significantly superior to the conventional system (copy-painting: 60.86 ± 6.56 bpm; free-painting: 58.93 ± 6.11 bpm). Subjective evaluations demonstrated significantly higher user satisfaction (4.10 ± 0.64 vs. 3.40 ± 0.75) and motivation scores (4.50 ± 0.51 vs. 2.85 ± 0.67), indicating enhanced operational efficiency and improved user experience. Although the means and standard deviations reported by the original authors provide preliminary evidence of the system's advantages, more comprehensive statistical analyses in future studies will be essential to rigorously validate these performance claims (Fig. 2e). Crucially, multimodal integration exhibits neuroplasticity across both spatial and temporal dimensions, further enhancing the robustness of neural decoding. Zhang et al. [39] designed a hybrid non-invasive BCI that integrates MI and high-frequency SSVEP (34/35 Hz) to control a wearable soft robotic glove designed for stroke rehabilitation. Methodologically, they employed the filter bank common spatial pattern (FBCSP) to process MI signals and the filter bank canonical correlation analysis (FBCCA) to decode SSVEP signals, ultimately making decisions through a weighted fusion algorithm. Online experimental results demonstrated the system's feasibility, achieving mean classification accuracies of 95.83% ± 6.83% in 12 healthy subjects and 63.33% ± 10.38% in 9 stroke patients. This high-frequency SSVEP reduced visual fatigue, while decomposed action imagery improved task intuitiveness, forming a closed-loop "peripheral-central-peripheral" rehabilitation framework. Meanwhile, although the dataset study by Lee et al. provided a theoretical foundation for the necessity of multimodal BCIs [101]. The study revealed that the MI paradigm had the highest rate of BCI illiteracy (53.7%), while the exogenous paradigms—ERP and SSVEP—exhibited much lower illiteracy rates of approximately 10%. More importantly, all participants were able to effectively control at least one paradigm, with no user being classified as universally illiterate. These results suggest that by developing adaptive systems that integrate multiple paradigms, it is possible to leverage the complementary strengths of different modalities, thereby extending control to a wider range of users (Fig. 2f).

In the domain of more complex passive BCIs and advanced cognitive state decoding, multimodal integration likewise demonstrates promise. Mirjalili and Duarte developed a transfer learning framework integrating EEG data from sustained attention [102], selective attention, and visual perception tasks to decode the encoding of episodic memory. Their findings suggested that tracking multidimensional cognitive states enhanced predictive validity through distributed neural engagement patterns, improving memory encoding prediction accuracy from 72% to 81.4% (*p* < 0.001). A more comprehensive understanding of the underlying neural mechanisms may be pursued in future work by leveraging complementary neuroimaging techniques (Fig. 2g). Kragel et al. [103] expanded this framework by demonstrating how affective states are spatially encoded across visual hierarchy regions (V1-PH), enabling decoding of complex cognitive-affective interactions (Fig. 2h). To address the challenges in multimodal integration and further enhance the performance of non-invasive BCIs, innovative deep learning networks will be a crucial future direction. Particularly, Transformer networks suitable for multimodal signal analysis, high-order feature fusion techniques, and algorithms based on cross-modal alignment will play a pivotal role.

### Multimodal Signal Acquisition: Techniques and System Design

EEG remains the foundational method for non-invasive BCI signal acquisition because of its high temporal resolution and non-invasive characteristics. However, EEG is susceptible to artifacts and has limited spatial resolution, which has led to growing interest in integrating multimodal neuroimaging techniques. Recent approaches combine EEG with complementary modalities: fNIRS provides hemodynamic data, while MEG enhances spatiotemporal resolution. This integration effectively addresses the inherent trade-off between temporal and spatial resolution in non-invasive systems [104–106].

**Fig. 3:**
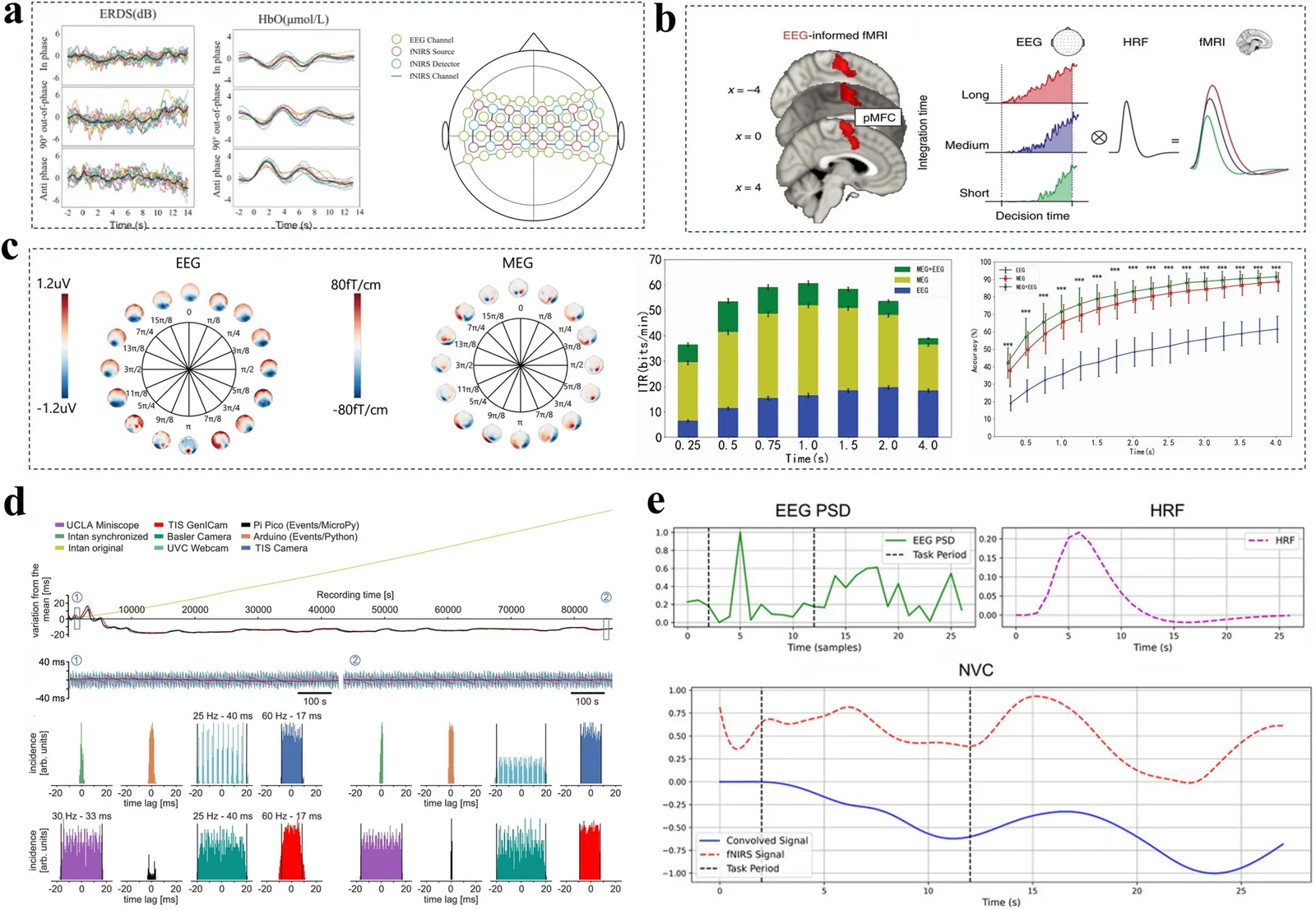
Schematic illustration of multimodal signal acquisition. **a** Left: EEG and fNIRS features calculated by TRCA. Right: EEG and fNIRS channels. Reproduced with permission [107]. Copyright 2022, IEEE. **b** EEG-informed fMRI and connectivity analyses in the value-based task. Reproduced with permission [108]. Copyright 2017, Springer Nature. **c** Left: 140 ms full-space event topography maps. Right: ITR and classification accuracy of single-modality EEG, single-modality MEG, and MEG-EEG fusion. Reproduced with permission [109]. Copyright 2024, IEEE. **d** Synchronization performance. Reproduced with permission [110]. Copyright 2017, Springer Nature. **e** Neurovascular coupling modeling. Reproduced with permission [111]. Copyright 2025, John Wiley and Sons

Recent studies in the field of BCIs have demonstrated that combining EEG and fNIRS can enhance system performance. Jiang et al. [107] developed a unified EEG-fNIRS bimodal signal processing framework to analyze single-trial neural signals during robot-assisted bimanual cyclical tasks. They employed the artifact subspace reconstruction algorithm to remove large-amplitude artifacts from EEG, and applied the temporal derivative distribution repair method to correct motion artifacts and baseline drift in fNIRS signals. The analysis time window was defined from 2 s before to 4 s after task execution, during which event-related desynchronization/synchronization (ERD/ERS) and oxygenated hemoglobin concentration changes (ΔHbO) were extracted as features. In terms of statistical methods, Jiang et al. found statistically significant differences in both ERD/ERS and ΔHbO responses among the three bimanual tasks during specific time intervals (*p* < 0.001), with post hoc analyses indicating that the anti-phase task elicited the strongest activation using statistical analysis. For classification, they used a support vector machine, which demonstrated that the fused EEG-fNIRS features achieved an accuracy of 90.1% in discriminating the three bimanual movement patterns—significantly higher than the accuracy using single-modality features (EEG: 74.8%; fNIRS: 82.2%) (Fig. 3a). FMRI offers extremely high spatial resolution, enabling precise localization of brain activity. However, its temporal resolution is constrained by the slow nature of the hemodynamic response. Simultaneous EEG-fMRI acquisition and fusion can combine the high temporal resolution of EEG with the high spatial resolution of fMRI, providing comprehensive spatiotemporal information about brain activity. This multimodal integration has been successfully applied in various BCI applications, including emotion recognition and MI. Pisauro et al. [108] employed a snack choice task combined with simultaneous EEG-fMRI and computational modeling to investigate the role of the posterior medial frontal cortex (pMFC) in value-based decision-making. To mitigate artifacts inherent to simultaneous recording, the study used twisted leads to reduce electromagnetic interference and performed offline removal of gradient artifacts. Results revealed that bold signal activity in the pMFC was significantly associated with these EEG dynamics, suggesting its role as a neural substrate for evidence accumulation during value-based choices (Fig. 3b). Ji et al. [109] constructed a 16-command SDMA-encoded MEG-EEG fusion modality BCI system. The study implemented a synchronous triggering mechanism via a 16-bit signal transmission link between visual stimuli and MEG data acquisition, ensuring precise synchronization between stimulus onset and data recording initiation. Without additional signal-domain preprocessing, the research team directly fused the two modalities and decoded them using the multiclass discriminative canonical pattern matching algorithm. Under a 4-s data window, the fusion model achieved an average classification accuracy of 91.71%, markedly exceeding that of MEG alone (88.57%) and EEG (60.76%), with corresponding p-values below 0.01 and 0.001. This performance gain was attributed to MEG's heightened sensitivity to contralateral polarity reversal in the occipital cortex, confirming the critical role of multimodal acquisition in enhancing spatial decoding performance in SDMA-based BCI systems (Fig. 3c). The Syntalos framework, developed by Klumpp et al. [110], is designed to address the challenge of precise synchronization in multimodal data acquisition, particularly for long-duration recordings. The framework established a globally shared master clock and continuously performs statistical analysis and correction of timestamps from various devices, ensuring precise alignment of all input timestamps. Experimental validation demonstrated that Syntalos maintains stable synchronization across devices for over 24 h. In a simulated time-drift experiment (with a systematic drift of 1 ms per second), they observed that the accuracy of the behavioral classifier dropped from nearly 100% to near-chance levels when the temporal misalignment between neural spike signals and whisker-touch behavioral data accumulated to 1 s. This demonstrated that Syntalos addressed the challenge of precise synchronization in multimodal data acquisition, particularly for long-duration recordings (Fig. 3d). Li et al. [111] proposed the BrainFusion framework to address challenges in reproducibility and deployment in multimodal BCI research. The framework manages signal temporal synchronization through standardized data containers and two alignment strategies (time-point alignment and event-based alignment), and supports the BIDS format to enhance data comparability. Case studies demonstrated its efficacy: it achieved 95.5% accuracy in EEG-fNIRS MI classification and deployed an EEG-ECG sleep staging model (80.2% accuracy) as an executable end application. The framework is primarily designed for offline analysis. Extending it to real-time closed-loop control constitutes a valuable direction for future research (Fig. 3e). The dual capacity to resolve spatiotemporal dynamics and endogenous modulation establishes multimodal approaches as critical for clinically viable BCIs [112]. To address multimodal engineering challenges, Bayesian frameworks and advanced algorithms have been widely applied in EEG source imaging to address source localization spatial priors [113]. To minimize mutual electromagnetic interference between sensors of different modalities, in addition to electromagnetic shielding techniques, signal processing filters tailored to specific noise characteristics should be integrated [84]. Moreover, in online or closed-loop systems, the phase lag inherent in signal transmission, processing, and actuator response cannot be ignored, as it directly determines the timeliness and effectiveness of closed-loop interventions. To compensate for such delays, advanced signal processing and predictive algorithms must be developed to optimize the efficiency of the processing pipeline and reduce computational complexity [114]. Time jitter and misalignment caused by systems with different sampling rates require coordinated advances in both precise hardware synchronization and software-level coordination [115, 116].

### Single-Model Deep Learning Architectures for Neural Dynamics Interpretation

Deep learning has become a key approach for decoding non-invasive neural signals [117–119], effectively addressing challenges such as spectral-temporal complexity, individual variability, and data scarcity. Although deep learning has achieved significant progress in enhancing decoding performance for non-invasive BCIs in recent years, its practical application still faces substantial challenges. These challenges represent the critical bottleneck in transitioning BCIs from laboratory settings to real-world deployment. So, an increasing number of recent studies have begun to actively simulate realistic conditions, introducing controlled perturbations to probe the performance limits of algorithms and thereby to reveal their applicability boundaries in real-world scenarios [120].

**Fig. 4:**
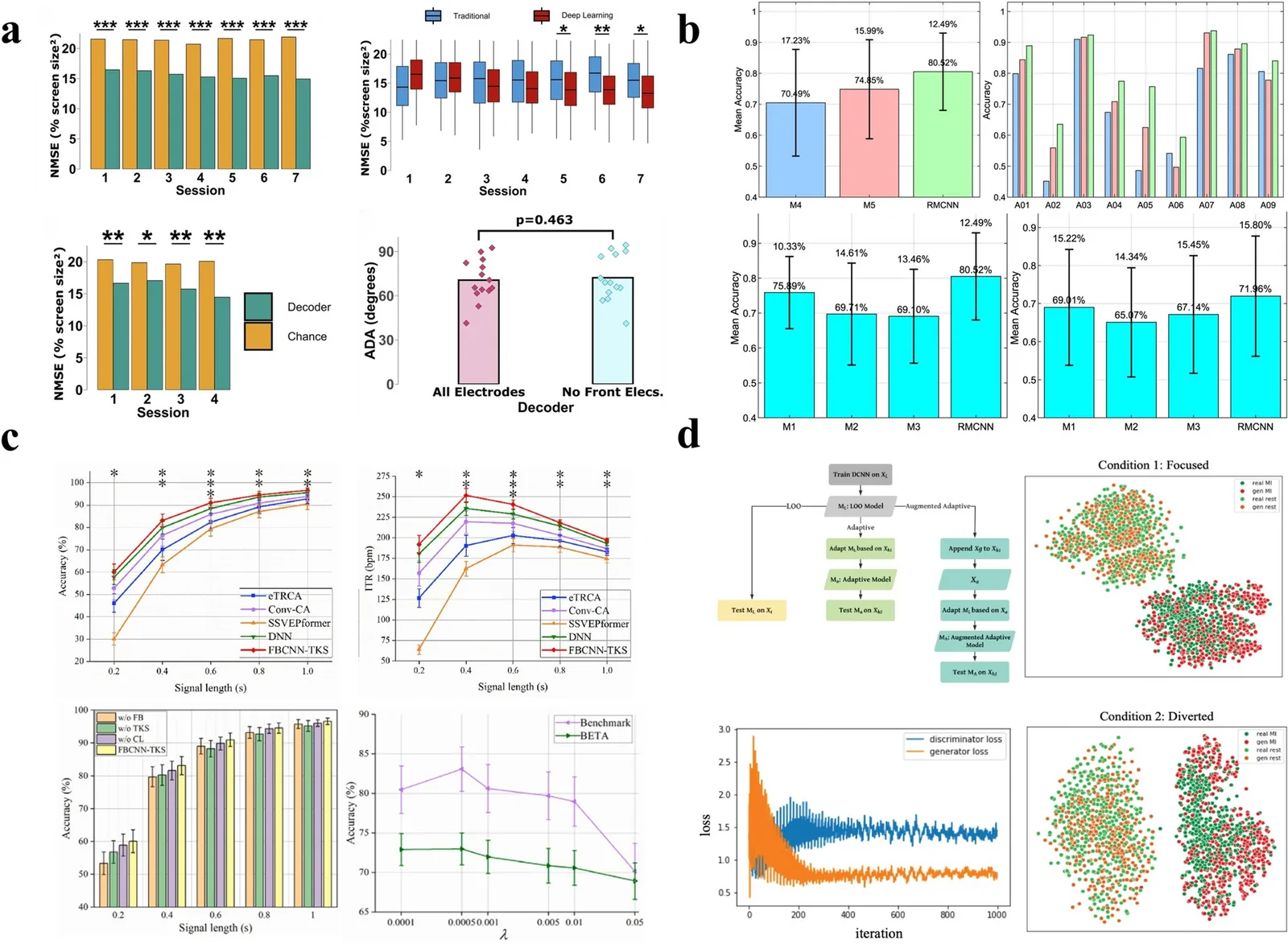
Schematic illustration of single-model deep learning architectures. **a** Comparison of BCI Decoder Performance with Random Levels and Traditional AR Methods. Reproduced with permission [121]. Copyright 2024, Springer Nature. **b** Top: Classification performance comparison of ablation studies on two datasets. Bottom: Classification performance comparison of models using different convolutional layer filter blocks. Reproduced with permission [123]. Copyright 2022, Elsevier. **c** Top: Accuracy of five models on the benchmark dataset. Bottom: Classification accuracy of the model under human intervention. Reproduced with permission [124]. Copyright 2025, Elsevier. **d** Left: Training loss of the generator and discriminator, and schematic of the model algorithm. Right: t-SNE visualization. Reproduced with permission [125]. Copyright 2021, IEEE

In terms of model architecture, researchers have explored diverse approaches centered around the spatiotemporal structure, manifold properties, and spectral patterns of EEG. Forenzo et al. [121] constructed an EEG-based BCI dataset comprising 28 subjects and 168 h of recordings, designed for online continuous pursuit tasks. Results demonstrated that deep learning (EEGNet, PointNet) decoders significantly outperformed chance level across all sessions. Forenzo et al. used a Holm-adjusted Wilcoxon signed-rank test to statistically validate this performance (*p* < 0.05), thereby supporting the reliability of MI features in the dataset. To systematically evaluate decoding stability under variations in electrode configuration and physiological noise interference, the authors removed five electrodes and introduced ocular artifacts into the data. Results showed no significant decline in decoding performance (*p* = 0.463), indicating the model's robustness in device variation scenarios. The model weights and code have been made publicly available (Fig. 4a). Li et al. [122] developed the HR-SNN model to investigate its robustness under noise interference and channel loss conditions. When subjected to Gaussian noise with an amplitude of 10%, the model achieved an accuracy of 68.59% using subject-specific transfer learning (SSTL). Under a more severe scenario with 16 out of 64 channels randomly removed, the SSTL accuracy remained at 71.48%.

In addition, Li et al. [123] proposed a Riemannian convolutional neural network (RMCNN) that integrates a spatial-temporal convolutional layer with a Riemannian block. The model was evaluated for cross-session generalization across three public MI datasets. Experimental results demonstrated that the model achieved an offline classification accuracy of 80.52% on the BCI Competition IV 2a dataset. To validate its effectiveness, Li et al. conducted a systematic comparison between RMCNN and baseline methods such as FBCSP and EEGNet using the Wilcoxon test, with statistical results confirming the significant advantages of their method. Furthermore, the study included an ablation experiment to assess the functional contributions of different components of the model. To promote research reproducibility, the authors have made the relevant code publicly available (Fig. 4b). Ju and Guan proposed Graph-CSPNet [96], which integrated graph neural networks with SPD manifolds for MI classification tasks. After establishing its excellent time-frequency feature extraction capability and classification effectiveness, they further enhanced model interpretability through spectral distribution visualization and graph Laplacian analysis. The approach clearly demonstrated the model's attention patterns toward different frequency bands in the time-frequency graph structure, including the *θ*, *μ*, *β*, and *γ* rhythms. The research team specifically emphasized that the frequency bands prioritized by their model show alignment with established neurophysiological mechanisms of MI. Subsequently, they introduced a geometric deep learning framework named Tensor-CSPNet [97]. This model represented EEG spatial covariance matrices on symmetric positive definite (SPD) manifolds and extracted spatio-spectro-temporal features of the signals by combining deep neural networks on SPD manifolds with CNNs. Under cross-session non-stationary scenarios, this model achieved an accuracy improvement of approximately 0.3 compared to FBCSP for Subject No.28 on the MI-KU dataset. Furthermore, the authors conducted a visualization analysis of the model's features using DeepLIFT and t-SNE. The results showed that the activation patterns captured by the model (such as contralateral activation in the C3/C4 regions) were consistent with the neurophysiological mechanisms of ERD/ERS during MI.

In addition to innovations in network architecture, training strategies and data augmentation methods also play a key role in performance improvement. The FBCNN-TKS model proposed by Huang et al. [124] demonstrated strong performance in offline analysis. With a 0.4-s data length, the model attained a classification accuracy of 83.10% and an ITR of 251.54 bpm on the Benchmark dataset, while achieving 72.98% accuracy and an ITR of 203.47 bpm on the BETA dataset. Huang et al. used a paired t test to validate the model's efficacy, with results confirming that FBCNN-TKS significantly outperformed traditional machine learning models as well as deep learning methods such as eTRCA and DNN (*p* < 0.01 on Benchmark, *p* < 0.001 on BETA). The study systematically validated the effectiveness of each model component through rigorous experiments: electrode configuration tests identified 9 or 32 channels as the optimal topology; ablation studies revealed that removing the filter bank, TKS module, or center loss consistently degraded performance, especially under shorter data lengths; and a hyperparameter sweep determined the optimal center loss weight *λ* to be 0.0005. These experiments collectively support the validity of the model architecture and the synergistic interactions among its components (Fig. 4c).

Fahimi et al. [125] proposed a framework based on conditional deep convolutional generative adversarial networks (DCGANs) to enhance training data by generating artificial EEG signals, addressing the challenge of degraded classification performance in BCI systems under diverted attention conditions. The work validated the diversity and realism of the generated signals through quantitative metrics, such as the closeness of the KL divergence between generated and real samples, and visualization analyses, including t-SNE embeddings and spectrogram comparisons. The adversarial training process demonstrated stability, with generator and discriminator losses converging after approximately 300 iterations, and no mode collapse was observed. In model evaluation, the method improved classification accuracy in the diverted attention condition from a baseline of 73.04% to 80.36%. To further assess generalization, the research team tested the model on the BCI Competition III Dataset IVa, where classification accuracy for different MI tasks increased from 67.57% to 71.14% (*p* < 0.02). These results collectively indicated that the proposed data augmentation strategy not only effectively enhanced BCI classification performance under diverted attention but also exhibited a degree of cross-dataset generalization capability (Fig. 4d). Zhang et al. [126] designed a multiple-source prototype-supervised adversarial transfer learning approach (PSAT). By constructing a multisource fusion framework that weighted integrates different source domains, PSAT reduced cross-subject discrepancies and intra-subject non-stationarity. It also addressed the challenges of cross-subject EEG classification in BCI. To rigorously validate the effectiveness of their method, Zhang et al. implemented a linear step-up procedure to control the false discovery rate, thereby mitigating the risk of false positives in multiple comparisons. Experimental results on three MI-EEG datasets demonstrated that PSAT achieved higher classification accuracy than its variants, PSAT-c, PSAT-a, and PSAT-v. Further ablation studies revealed that both the prototype mapper and domain discriminator were indispensable modules for enhancing system performance.

**Fig. 5:**
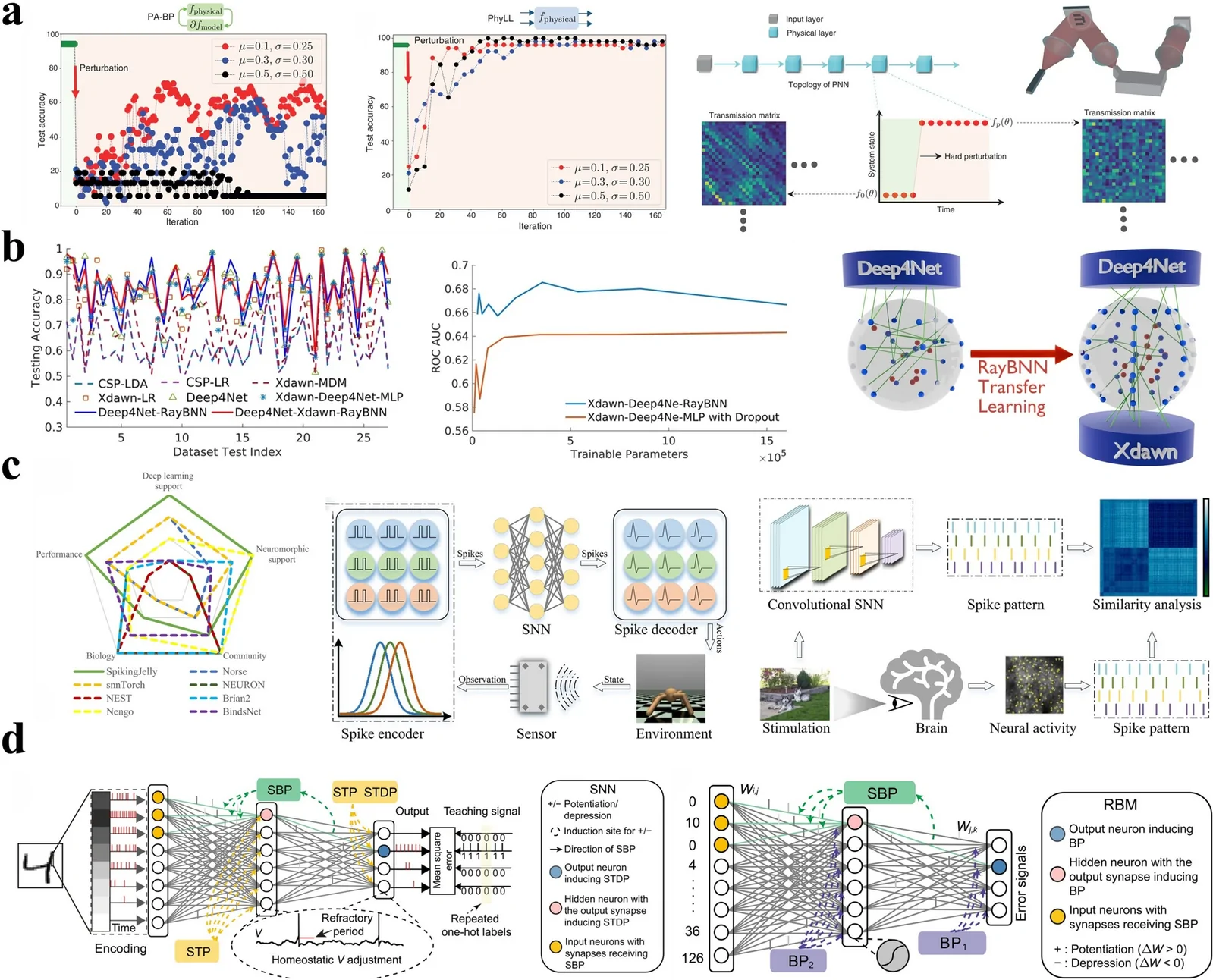
Schematic illustration of single-model deep learning architectures. **a** Robustness of deep PNNs against unpredictable external perturbations. Reproduced with permission [127]. Copyright 2023, The American Association for the Advancement of Science. **b** Transfer learning algorithms on the EEG dataset. Reproduced with permission [128]. Copyright 2024, Springer Nature. **c** Left: Comparisons of the Spiking Jelly with other frameworks and applications. Right: Typical applications of Spiking Jelly. Reproduced with permission [129]. Copyright 2023, The American Association for the Advancement of Science. **d** Introducing biological SBP into SNNs. Reproduced with permission [130]. Copyright 2021, The American Association for the Advancement of Science

In addition to the aforementioned models, Momeni et al. [127] proposed the physical local learning (PhyLL) algorithm, establishing a paradigm for training physical neural networks. To evaluate the robustness of the system, the original authors introduced Gaussian noise perturbations (mean *μ* = 0.1–0.5, standard deviation *σ* = 0.25–0.50) into the optical system. Under severe perturbations caused performance degradation, PhyLL recovered high accuracy within just a few training epochs. In contrast, the digital model-dependent physics-aware backpropagation (PA-BP) method exhibited its accuracy decline to approximately 55% even under mild perturbations and failed to recover. After validation on multiple physical platforms, this work demonstrated the adaptive capability of physical neural networks in unstable environments and laid the groundwork for future exploration of their potential in BCIs to address challenges such as channel characteristic variations and environmental interference (Fig. 5a). Yuen et al. [128] designed a three-dimensional ray-traced biological neural network (Ray-BNN), demonstrating potential in transfer learning for dimension-varying tasks. The model preserved trained weights and supported architectural adaptation during network expansion through dynamic 3D neuronal connectivity and sparse matrix representations. On an EEG dataset comprising multiple paradigms, Ray-BNN achieved an accuracy of 85.6% (*p* ≤ 1.7968 × 10⁻^3^) in 54-fold subject-independent testing by integrating feature extractors from Deep4Net and X-dawn, indicating robust cross-subject generalization. During transfer learning on the Alcala dataset, input dimensions progressively increased from 6 to 162 access points. The model reduced cumulative training time by approximately 85% compared to BiLSTM while maintaining sparsity below 40% in the weight matrix. The study provided publicly available code and data to support reproducibility. The foundation for cross-subject generalization established by this study provides a clear direction for future work: verifying the stability of transfer performance in scenarios with device heterogeneity will drive substantial advancements of the framework in complex application environments (Fig. 5b). Spiking neural networks (SNNs), as pivotal enablers of brain-inspired intelligence, have recently seen significant advances in both software tools and learning algorithms. At the framework level, the Spiking Jelly framework introduced by Fang et al. [129] has effectively bridged a critical gap in the dedicated software toolchain for spiking deep learning. Spiking Jelly significantly reduced the technical barrier to SNN research and promotes the development of the software ecosystem for neuromorphic computing (Fig. 5c). At the algorithmic level, the biologically inspired self-backpropagation (SBP) mechanism proposed by Zhang et al. [130] facilitates coordinated weight adjustment in both SNNs and ANNs by allowing synaptic modifications (potentiation or depression) at output neurons to backpropagate across layers to upstream synapses (Fig. 5d). Current research on deep learning-based decoding for non-invasive BCIs is evolving from singular performance optimization toward building multidimensional capabilities. Architecturally, models are becoming more aligned with the spatiotemporal and geometric properties of neural signals. In training, methods increasingly integrate data augmentation, transfer learning, and alignment strategies to enhance generalization. Nevertheless, there remains a notable absence of exploration into performance boundaries under real-world conditions such as channel loss, noise interference, and attentional fluctuations. Most approaches remain focused on offline analysis, and the generalization limits in dynamic environments and with device heterogeneity require more systematic evaluation, which represents a critical direction for future research.

### Hybrid Deep Learning Frameworks for Multiscale Signal Decoding

Single-model decoding approaches, though prevalent, are often limited by their generalizability, especially when faced with complex, real-world data. In contrast, hybrid architectures have emerged as a promising solution by integrating spatial, temporal, and spectral representations. These approaches effectively address key challenges such as cross-trial variability and signal complexity, setting the stage for robust decoding in both motor and linguistic domains.

**Fig. 6:**
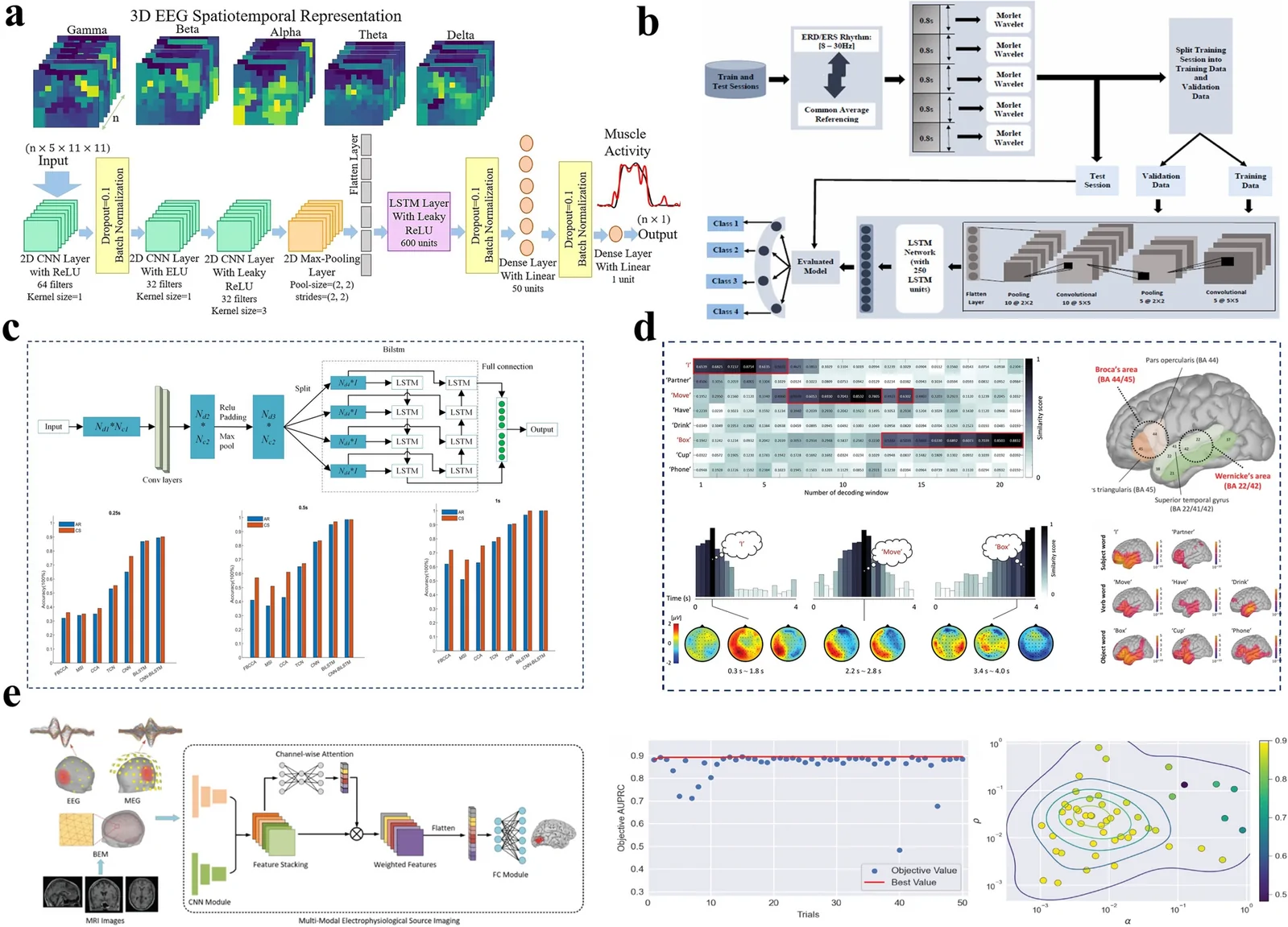
Hybrid deep learning frameworks for multiscale signal decoding. **a** Architecture of the CNN-LSTM. Reproduced with permission [131]. Copyright 2025, Elsevier. **b** Architecture of CNN-LSTM. Reproduced with permission [132]. Copyright 2022, Elsevier. **c** Architecture of CNN-BiLSTM network and recognition accuracy for different methods. Reproduced with permission [133]. Copyright 2024, Elsevier. **d** Decoding process of deep neurolinguistic learning in real time. Reproduced with permission [134]. Copyright 2023, IEEE. **e** Multimodal deep fusion framework and hyperparameter tuning using Bayesian optimization. Reproduced with permission [135]. Copyright 2024, IEEE

In the area of motor decoding and electromyographic activity prediction, existing methods often struggle to capture the nonlinear relationship between EEG and EMG signals. Therefore, developing nonlinear models capable of integrating spatiotemporal features, such as the CNN-LSTM model, is crucial for extracting muscle activity-related information. Amiri and Shalchyan proposed a deep learning model integrating CNN and LSTM networks to decode muscle activity from non-invasive EEG signals [131]. During the grasp and lift (GAL) task, their model achieved an average correlation coefficient (CC) of 0.76 ± 0.10 and a normalized root mean square error (nRMSE) of 0.21 ± 0.05 between the actual and predicted muscle activities for two muscles. They employed statistical tests with Bonferroni correction (adjusted *p*-value < 0.016) confirmed that the model significantly outperformed two comparative methods: multivariate linear regression (mLR) and multilayer perceptron (MLP). Building on these promising results, several aspects emerge as valuable avenues for further investigation. For instance, while the current evaluation was conducted offline, exploring real-time implementation represents a natural and important next step, particularly considering the computational profile of the model. The average decoding time for the CNN-LSTM architecture was 339.76 ± 51.71 s. This provided a useful baseline for future work aimed at optimizing efficiency for real-time applications, especially when compared to the faster mLR (3.93 ± 3.02 s) and MLP (95.40 ± 22.44 s) models (Fig. 6a). Although CNN-LSTM can automatically extract spatiotemporal features, it requires large amounts of data for training. To address the issue of data scarcity, Khademi et al. [132] proposed a hybrid CNN-LSTM deep learning model based on transfer learning and data augmentation, aiming to enhance the classification performance of MI-EEG signals. Their approach employed the continuous wavelet transform (CWT) to convert EEG signals into time-frequency images. They expanded the dataset fivefold (from 288 to 1440 trials per subject) using a non-overlapping sliding window cropping strategy. Evaluated on the BCI Competition IV dataset 2a, this model achieved a mean classification accuracy of 86% and a mean Kappa value of 81% (Fig. 6b).

In the direction of rehabilitation systems. An et al. [133] developed a real-time classifier based on CNN-BiLSTM for a patient-centered AR-SSVEP active rehabilitation exoskeleton system. The study achieved an offline classification accuracy of 98.5% and an ITR of 210 bits min^−1^ within a 0.5-s time window. In the online experiments, the subjects took an average of 319 s to complete four non-repetitive trajectory tasks, which was 20% longer than the standard reference time. As an initial investigation involving six healthy young participants, this study established a foundation for subsequent research aiming to expand subject diversity (Fig. 6c).

In the field of neural language decoding and interactive control. Jeong et al. [134] proposed a real-time, non-invasive neural language decoding method based on deep neurolinguistic learning for multiuser BCI. This approach combined a CNN and a gated recurrent unit (GRU) to decode speech imagery from EEG signals. It then integrated the decoded results into sentence-level neural commands via a rule-based sentence generation model to control a neural prosthetic arm. The study designed multiuser collaborative scenarios, allowing different users to control the prosthetic arm for themselves or their partners. The decoded neural language is used to perform high-level tasks such as object delivery and emotional interaction. The overall average task success rate was 72.36%, with an average latency of 4.450 s per word and 8.645 s for sentence generation (Fig. 6d).

In the domain of multimodal fusion and electrophysiological source imaging. Jiao et al. [135] proposed a multimodal deep fusion framework using attention neural networks (MMDF-ANN). This framework aimed to improve localization accuracy and stability under conditions of extended sources and low SNR in electrophysiological source imaging (ESI). The framework used dual-stream CNN modules to process EEG and MEG signals separately and integrated the features through a channel-wise attention mechanism. On synthetic data, the model demonstrated strong performance. For instance, in single-source localization tasks with an SNR of 30 dB, it achieved an area under the precision-recall curve (AUPRC) of 0.944 ± 0.104, outperforming traditional methods such as MNE and sLORETA, as well as the unimodal deep learning model ConvDip. Ablation studies confirmed that both multimodal fusion and dilated convolution contributed to the performance improvement. Notably, as the number of sources increased, the model's performance exhibited a declining trend: in four-source tasks, the AUPRC dropped to 0.827 ± 0.166. This phenomenon suggested that the diversity of training data may affect the model's adaptability to complex activation patterns. In validation with real data, MMDF-ANN successfully localized visual cortex activity in a face perception dataset containing simultaneous EEG/MEG recordings. In an epilepsy dataset with only EEG recordings, the model still achieved more focal source localization compared to ConvDip. These findings establish a solid foundation for multimodal fusion approaches, while their broad applicability in diverse clinical environments represents a promising direction for future validation (Fig. 6e).

Future work could adopt domain adaptation methods to improve device compatibility and establish comprehensive performance degradation curves to better define the algorithm's operational boundaries. Together, these methodological advances illustrate the considerable potential of hybrid deep learning strategies that integrate multiscale feature encoding and cross-modal alignment [136]. While significant gains have been demonstrated across decoding tasks and modalities, future work must address cross-subject generalizability and robustness in real-world environments [85, 137].

### Toward Real-Time, Asynchronous, and Collaborative Non-Invasive BCIs

**Fig. 7:**
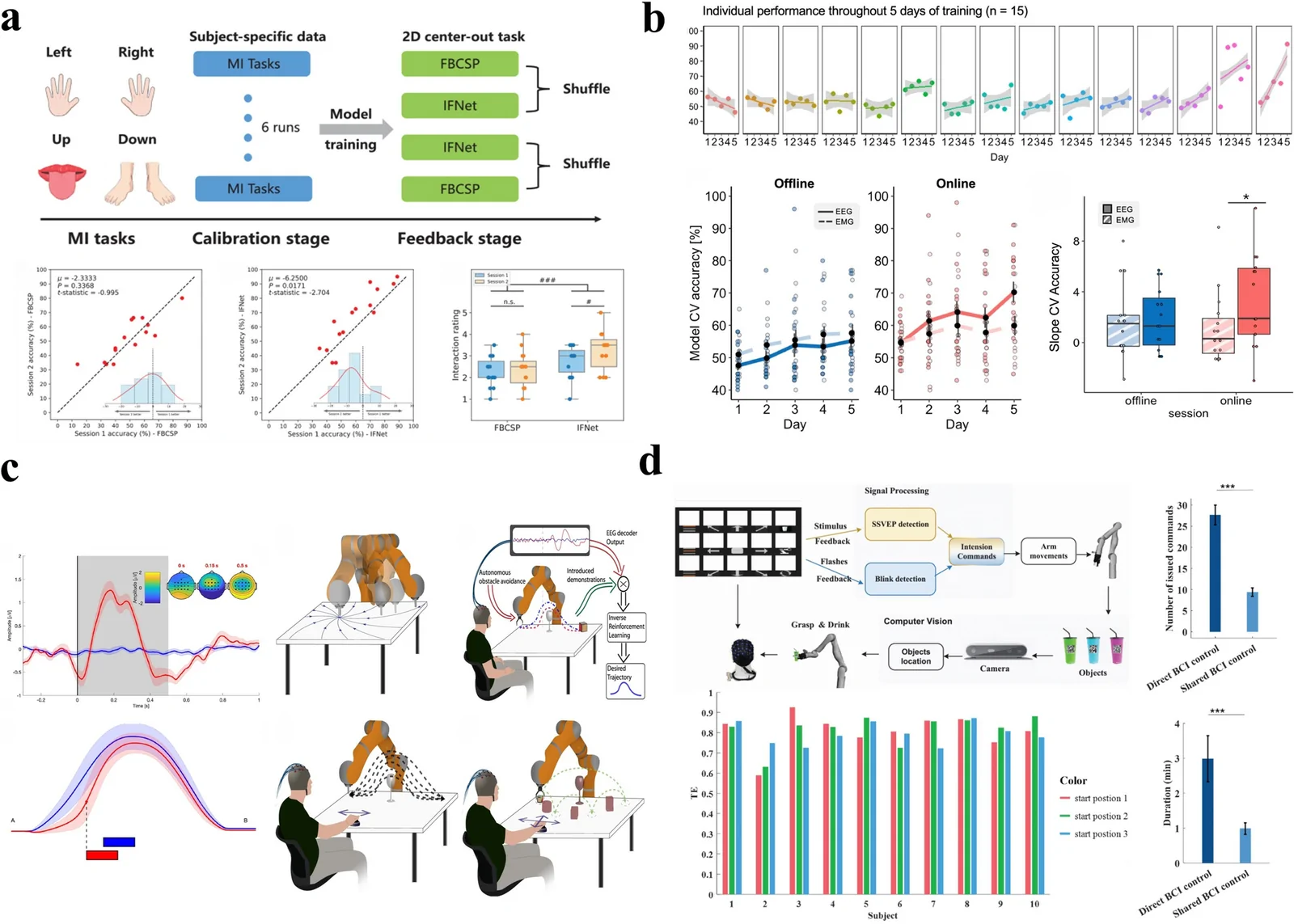
Schematic illustration of real-time, asynchronous, and collaborative non-invasive BCIs. **a** Top: Schematic illustration of MI-BCI experimental paradigm. Bottom: Comparative performance of online BCI control. Reproduced with permission [138]. Copyright 2025, IEEE. **b** Decoding based on EMG signals. Reproduced with permission [139]. Copyright 2025, Springer Nature. **c** Left: ErrP decoding results. Right: Schematic of the control architecture and experimental protocol. Reproduced with permission [140]. Copyright 2021, Springer Nature. **d** Left: Schematic of the BCI robot control system and TE of the robot 3D reach task. Right: Comparison of the strategies of direct BCI control and shared BCI control. Reproduced with permission [142]. Copyright 2023, IEEE

There is a growing focus in non-invasive BCI development on real-time performance, shared control strategies, and asynchronous detection capabilities. Wang et al. [138] conducted a randomized cross-session online study to evaluate the performance of the deep learning model IFNet in 15 BCI-naive subjects. Experimental results demonstrated that IFNet significantly outperformed the traditional method, FBCSP, in both sessions. The model achieved improvements in online task accuracy of 20% and 27%, respectively, and the main effect of the model was significant (*P* < 0.0001). Methodologically, the original authors used Bonferroni correction for all statistical tests involving multiple comparisons, ensuring the rigor of statistical inference. Furthermore, they adopted the CutMix data augmentation strategy, which was confirmed to enhance model performance (*P* < 0.05). Ablation experiments further validated the effectiveness of this strategy across multiple datasets. Additionally, the authors made the complete model code publicly available and reported an inference latency of approximately 5 ms, demonstrating the potential of this method for practical deployment. Notably, the study also revealed key limitations of the model: offline analysis showed that when the model was transferred from calibration tasks to online feedback tasks, significant performance degradation occurred in both cross-session and cross-mode scenarios; sensitivity analysis by the original authors further indicated that when the decision window was shorter than 1 s, the model decoding accuracy dropped sharply, even below the chance level (Fig. 7a). In another study focusing on training effects, Bhadra et al. [139] employed a rigorous experimental design in a 5-day EEG-based imagined speech BCI study, revealing the mechanisms by which training enhances real-time control capabilities. Results demonstrated that after receiving continuous feedback training, 15 healthy participants significantly improved their BCI control accuracy from 55 to 70% (*p* = 0.018), with online cross-validation accuracy significantly surpassing the offline condition (*T*_14_ = 8.3, *p* = 8.8 × 10^-7^, *d* = 2.14). Pronounced individual differences were also observed, as indicated by a significant positive correlation between learning slopes and average performance (*r* = 0.55, *p* = 0.034), corroborating the prevalence of the "BCI illiteracy" phenomenon in imagined speech tasks. At the neural mechanism level, the study identified power enhancements in the frontal theta band and the left temporal low-gamma band as key biomarkers of learning. Technically, the system achieved an average feedback delay of 100 ms, and the code and model weights were made publicly available. Notably, although EMG signals showed some contribution to offline decoding (EMG vs. EEG: *T*_14_ = 2.2, *p* = 0.044, *d* = 0.57), online EEG decoding accuracy was significantly higher (*T*_14_ = 2.77, *p* = 0.014, *d* = 0.71) (Fig. 7b).

Regarding shared control paradigms, Batzianoulis et al. [140] developed a BCI-based shared control architecture that integrates the autonomous obstacle avoidance capability of a robot with users' implicit neural feedback. In this framework, the robot generated real-time trajectories through a dynamical system, while users could exercise a "veto" against trajectories that did not align with their personal preferences by eliciting error-related potentials (ErrPs). Batzianoulis et al. employed rigorous statistical testing to validate this paradigm's effectiveness: they adopted repeated-measures ANOVA, which revealed a significant difference in ErrP decoding accuracy between the time-locked and continuous modes (*F* (1, 12) = 27.1, *p* < 0.001), with online continuous decoding maintaining an accuracy of 70 ± 13%. Building upon this, posterior probability-weighted inverse reinforcement learning significantly reduced the frequency of user interventions after only 3–5 demonstrations (*p* < 0.001). The progress made in this shared control system, particularly in trajectory distribution reporting, highlights key areas for future breakthroughs. By precisely quantifying trajectory errors and establishing clear human-machine decision boundaries, future work could substantially improve the reliability and safety of shared control paradigms in practical applications (Fig. 7c). Similarly, focusing on shared control, Deng et al. [141] proposed a self-adaptive shared control method based on a brain state evaluation network (BSE-NET) for human-wheelchair cooperation. This system evaluated the user's brain control ability online via quantized attention-gated kernel reinforcement learning and generated a confidence score to dynamically adjust the control weight between robot autonomy and human operator. Experimental results demonstrated that most subjects achieved a task success rate of approximately 90% in dynamic environments, and a significant correlation was observed between the confidence score and EEG decoding accuracy in online experiments. In a complementary approach, Lu et al. [89] proposed a model predictive control (MPC)-based shared control method. By explicitly setting safety constraints (such as lateral and yaw angle error bounds) and introducing a penalty term (weight *α* = 1) for deviations from the driver's command within its optimization framework, this method delineated a quantifiable human-machine trade-off space at the system design level. This approach significantly improved task performance: in obstacle avoidance tests, direct brain-controlled driving failed. However, with MPC assistance, some participants achieved a 100% task success rate, while others attained success rates between 85% and 95%. Future work could focus on conducting multisession, long-term experiments to systematically investigate the system's adaptive capabilities in dynamic environments. This approach would facilitate the evaluation of the framework's performance sustainability under realistic operational conditions.

Taking a step further, Zhou et al. [142] created a shared control system for a 3D robotic arm that integrates a hybrid asynchronous BCI (converging SSVEP and electrooculography (EOG) signals) and computer vision. The system adopted a 15-command asynchronous interface and a 3D vector synthesis strategy to enable flexible manipulation of the robotic arm in unknown environments. Online experiments demonstrated that in a free spelling task, the system achieved an average classification accuracy of 92.4% ± 5.5%, a false positive rate of 1.25% ± 0.71%, and an ITR of 97.9 ± 12.8 bits min^−1^. In the 3D reach-grasp-drink task, the shared control mode significantly reduced the number of commands issued (9.40 ± 2.14 vs. 28.90 ± 6.83), the BCI-guided phase completion time (0.99 ± 0.35 vs. 3.04 ± 1.42 min), and the number of error commands (0.35 ± 0.59 vs. 2.25 ± 2.02) compared to direct BCI control. The average trajectory efficiency was 0.80 ± 0.10. They used the NASA Task Load Index (NASA-TLX) to assess user cognitive load (scores for most dimensions < 30), though mental demand and effort scores were relatively higher. Future research could focus on quantifying the cumulative risks of false positives and false negatives during extended usage periods (ranging from several hours to multiple days). Additionally, providing complete distribution data for trajectory errors and task completion rates would offer valuable insights for validating system stability and broadening its practical applications (Fig. 7d). On the engineering implementation front of asynchronous BCIs, Hu et al. [143] developed a wearable asynchronous BCI system based on EEG and EOG signals. The system utilized a self-developed compact amplifier and required only three EEG channels (Cz, P3, P4) and one EOG channel. It achieved asynchronous control through the integration of P300 potential detection and blink recognition. In a telephone dialing task, the system attained an average online accuracy of 94.03% ± 4.65%, an ITR of 31.42 ± 7.39 bits min^−1^, and a low false positive rate of 1.78% ± 2.25% during a 10-min idle state. These promising results provide a solid foundation for future investigations into the system's long-term performance characteristics, particularly regarding the cumulative risk profiles of false positives and false negatives during extended multiday usage scenarios. In another asynchronous BCI study, Aloise et al. [144] designed an asynchronous BCI system based on ERPs. Online experimental results showed that the correct classification rate of the asynchronous classifier (74.66%) was slightly lower than that of the synchronous classifier (87.96%), and the error rate (7.11%) was also lower than that of the synchronous classifier (12.04%). However, neither difference was statistically significant (error rate: *p* = 0.19). The asynchronous classifier demonstrated good robustness during the no-control state, with an average false positive rate of 0.16 per minute (i.e., fewer than one false positive every six minutes). The asynchronous BCI based on SSVEPs designed by Gernot R. [145]. The participants took 75.5 to 217.5 s to complete the movement sequence. The number of false negative decisions varied from 0 to 10 (with a maximum possible of 34 decisions).In the clinical application of BCIs, Alcaide-Aguirre et al. [146] developed an asynchronous BCI based on the P300 ERP to facilitate the administration of the Peabody Picture Vocabulary Test (PPVT-IV) for cognitive assessment in individuals with cerebral palsy. Results from the NASA-TLX demonstrated that participants with CP perceived the BCI-facilitated PPVT-IV as significantly higher in mental demand, physical demand, and effort (*p* < 0.05) compared to their typically developing counterparts.

In summary, current research has made significant progress in enhancing BCI real-time performance, implementing intelligent shared control, and constructing robust asynchronous systems. However, a common, unresolved core issue persists: the vast majority of systems lack an assessment of their stability and reliability under conditions of prolonged (several hours) and multiday continuous use. This is specifically reflected in the failure to adequately quantify the cumulative risk of false positives and false negatives, the inability to strictly define the human-machine trade-off boundaries in shared control based on complete data distributions (e.g., trajectory error, task completion rate), and the lack of systematic analysis of the long-term impact of control parameters (such as penalty weights in model predictive control). Addressing these issues is crucial for advancing non-invasive BCIs from the laboratory to real-world applications.

### AI-Enabled Human-Machine Co-Adaptive Systems

**Fig. 8:**
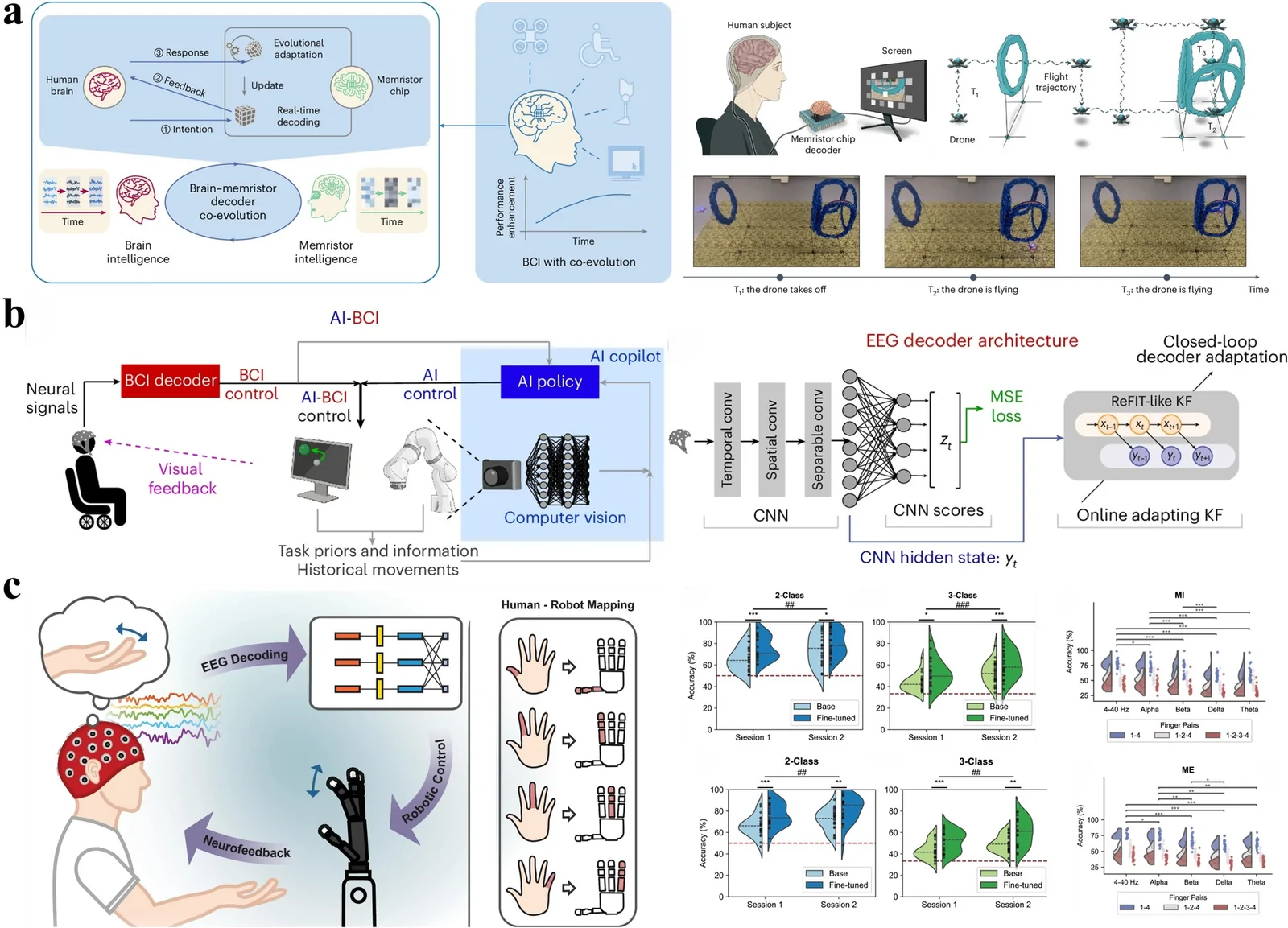
Schematic illustration of AI-enabled human-machine co-adaptive systems. **a** Left: Schematic diagram depicting the human-machine joint learning process and the experimental paradigm. Right: Illustration of BCI training with different strategies. Reproduced with permission [148]. Copyright 2025, Springer Nature. **b** Left: AI copilots use task information to improve BCI performance. Right: CNN-KF architecture. Reproduced with permission [149]. Copyright 2025, Springer Nature. **c** Left: Experimental paradigm. Middle: Effects of online training on MI and ME robotic finger control. Right: ME and MI tasks using EEG signals filtered with different bandpass settings. Reproduced with permission [150]. Copyright 2025, Springer Nature

Particularly crucially, current BCI research is undergoing a shift from "open-loop, static" systems to "closed-loop, adaptive" paradigms [92], with its development direction focusing on enhancing real-time decoding capabilities, improving asynchronous detection mechanisms [37], and optimizing shared control strategies [147]. Extending the principle of co-adaptation to resource-constrained settings, Liu et al. [148] developed a neuromorphic decoding SSVEP of BCIs based on a 128,000-cell memristor chip. Their approach featured an interactive update framework that enabled co-evolution between the memristor decoder and the user's dynamic brain signals. This architecture condensed the traditional three-stage pipeline of preprocessing, feature extraction, and classification into a hardware-friendly one-step operation, reducing computational complexity by approximately 6.5 times while maintaining software-equivalent accuracy. In a brain-controlled drone task with four degrees of freedom, the system demonstrated significant improvements in energy efficiency and processing speed: decoding consumed 1,643,000 times less energy and achieved a 216-fold increase in normalized throughput compared to a CPU-based system. A closed-loop co-evolution framework driven by ErrPs enhanced decoding accuracy by approximately 20% during a six-hour online experiment involving 10 participants, establishing a foundational benchmark for low-power, long-term adaptive BCI operation (Fig. 8a).

Building on this foundation, Lee et al. [149] proposed a hybrid adaptive decoding approach and developed a shared autonomy-based BCI system that enhanced performance by introducing an AI copilot. This framework used a CNN to extract complex nonlinear features, which were then passed into a Kalman filter (KF) to predict and correct the user's movement intent. Additionally, the system integrated closed-loop decoder adaptation (CLDA), enabling dynamic optimization of decoding parameters. Experimental results demonstrated that the cursor control system significantly improved task efficiency. The average target acquisition rate increased by 2.1-fold in healthy participants and 3.9-fold in a paralyzed participant, with optimized movement trajectories. In the robotic arm sequential pick-and-place task, the paralyzed participant could not complete the task without the AI copilot but achieved a 93% success rate with its assistance. Future research could focus on the following promising directions: developing adaptive decoding algorithms that accommodate individual variability and EEG non-stationarity, exploring autonomous perception and decision-making mechanisms for environments with unknown targets or dynamic conditions, and validating the system's generalization capabilities in more complex application settings. These research avenues would facilitate the transition of BCIs from controlled laboratory environments to practical real-world applications (Fig. 8b). Ding et al. [150] proposed a deep learning model based on the EEGNet architecture, combined with a fine-tuning mechanism, facilitating non-invasive real-time decoding of individual finger MI and motor execution (ME) for robotic hand control. Among 21 able-bodied subjects with BCI experience, the system achieved notable results. Online decoding accuracies reached 80.56% for two-finger MI tasks and 60.61% for three-finger MI tasks, while the corresponding ME tasks achieved 81.10% and 60.11%, respectively, indicating highly comparable control performance between the two paradigms. Ding et al. confirmed through cross-session analysis that the model's online accuracy significantly improved with training (two-finger MI: *F* = 7.127, *p* < 0.001), and applied Bonferroni correction in subsequent post hoc analyses to confirm the statistical significance. The study also revealed clear performance boundaries: four-finger classification accuracy remained only approximately 46%, insufficient for practical application, while the index and middle fingers exhibited the lowest decoding performance due to highly overlapping neural representations. In the model interpretability analysis, they demonstrated through group-averaged saliency topological maps that the brain regions prioritized by the models exhibit spatial consistency across different subjects. This indicated that the decision-making patterns were grounded in neural activity features shared across the population. The establishment of an adaptive validation framework for complex dynamic environments represents a critical breakthrough for future research (Fig. 8c). To expand control granularity, in the realm of semantic neural decoding, Tang et al. [37] proposed a fMRI-based language reconstruction framework that integrates a generative pre-trained transformer model with beam search to decode continuous natural language. The system bridges internally generated cognition, such as mental imagery, with stimulus-driven conditions, including speech or video perception, achieving a peak contextual semantic similarity score of 0.8116 (*q* < 0.05) across diverse input modalities. Despite its high performance, decoding accuracy significantly deteriorated when participants engaged in distraction tasks. These empirical findings highlight the decoder's sensitivity to user attention and cognitive state, underscoring the critical importance of behavioral context in human-machine co-adaptive systems.

In summary, AI is driving BCIs toward human-machine co-adaptive systems capable of dynamic, personalized interaction [6, 151]. From hardware efficiency to semantic decoding, recent advances demonstrate appreciable potential. Yet, challenges in individual variability, environmental robustness, task dependency, and cognitive state awareness continue to hinder scalability and real-world applicability. Future progress will depend not only on better models, but on building intelligent ecosystems that are context-aware, continuously learning, and capable of seamless cross-platform integration.

## Integration of Flexible Bioelectronics in Non-Invasive BCIs

In contrast to invasive BCIs, which insert probes directly into target tissue, non-invasive BCIs monitor macroscopic electric fields outside the cranium to acquire EEG signals. Although this approach sacrifices spatial resolution and signal quality, it avoids the tissue damage caused by invasive techniques and potential long-term neuroinflammation. Furthermore, it facilitates rapid and widespread application without the need for complex surgical implantation. These advantages have secured long-standing and broad interest in non-invasive BCIs [12, 14]. However, their wearable nature presents numerous challenges for EEG acquisition. Firstly, the contact impedance between the electrode and the biointerface significantly impacts signal quality. Reducing this impedance can be approached from two angles: enhancing the electrode's intrinsic conductivity and ensuring conformal contact at the biointerface. Consequently, compared to traditional rigid electrodes, flexible electrodes not only improve signal quality but also offer greater wearing comfort, facilitating long-term monitoring. Flexible electrodes can be fabricated from intrinsically conductive thin-film materials, such as certain organic semiconductors, or by integrating conductive nanomaterials with a flexible substrate [152]. Particularly, driven by innovations in materials science, nanomaterials are receiving growing interest and application in flexible electrodes. This synergy between the distinctive electrical properties of nanomaterials and the favorable mechanical flexibility of substrates provides a robust foundation for high-performance flexible electrodes [151, 153, 154].

For instance, nanowires (NWs), as one-dimensional materials, possess a high aspect ratio. This structure endows them with excellent mechanical flexibility, allowing them to maintain structural stability when conforming to irregular surfaces or under bending conditions [153, 155]. Concurrently, due to their efficient electrical transport capabilities, they serve as conductive fillers that significantly enhance the electrode's conductivity [156]. This has been associated with reduced contact impedance and improved SNR in non-invasive BCIs. Furthermore, mechanical stability plays a key role in non-invasive BCIs. Superior mechanical properties contribute to achieving conformal contact with the target surface, promoting wearing comfort, and maintaining performance stability during long-term monitoring while reducing the risk of degradation [152]. Due to their nanoscale dimensions, nanomaterials exhibit excellent robustness. They could maintain their performance under repeated strain, unlike bulk materials that suffer from performance decay due to mechanical fatigue. It is noteworthy that artifact contamination in non-invasive BCI recordings, especially during target movement, complicates EEG interpretation. These artifacts are generally attributable to two origins: biological signals and non-biological signals. Non-biological artifacts are largely motion-induced, resulting from electrode shift that compromises stable interfacial contact. This instability manifests as variable contact impedance and introduces artifacts. The integration of nanomaterials into flexible substrates presents a promising strategy for ameliorating this challenge. In summary, innovations in materials science are essential for achieving higher performance in non-invasive BCIs, and nanomaterials represent a promising candidate for this pursuit.

### Flexible Conductive Thin Films: Materials Innovation and Interface Optimization

**Fig. 9:**
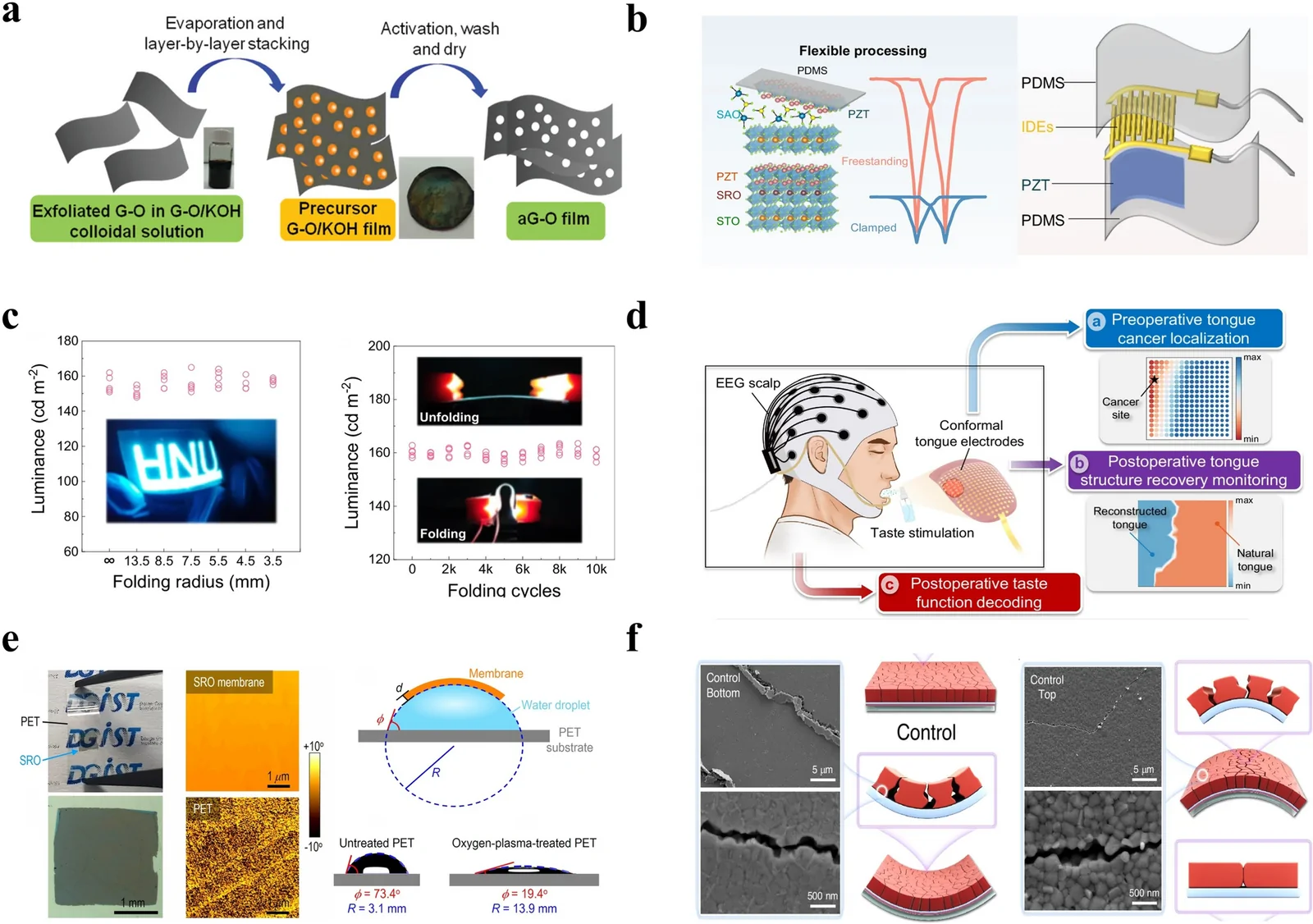
Schematic illustration of the performance characteristics of flexible conductive films. **a** Schematic diagram of the preparation process for aG-O films. Reproduced with permission [64]. Copyright 2012, American Chemical Society. **b** Detailed construction of the flexible PENG device. Reproduced with permission [159]. Copyright 2025, Springer Nature. **c** Left: A plot of luminance for a foldable ACEL device. Right: A plot of the luminance stability of it undergoing a cyclic folding test. Reproduced with permission [160]. Copyright 2024, Springer Nature. **d** Schematic overview of the gustatory interface. Reproduced with permission [161]. Copyright 2024, Springer Nature. **e** Left: Flexible single-crystalline SRO membranes. Right: Stress relaxation of the film on a water droplet. Reproduced with permission [162]. Copyright 2022, Springer Nature. **f** Bending durability of flexible perovskite films. Reproduced with permission [163]. Copyright 2025, Springer Nature

The development of non-invasive BCIs is closely linked to advancements in flexible conductive materials that strike a balance between electrical properties, mechanical durability, and biocompatibility. Recent advances in materials science indicate that conductive films offer viable pathways for overcoming persistent challenges in signal fidelity, motion artifact suppression, and long-term wearability [157, 158]. For instance, Zhang et al. [64] developed activated reduced graphene oxide (aG-O) films exhibiting in-plane conductivity of 5,880 S m^−1^. These films exhibit exceptional charge transport properties, which are crucial for preserving signal fidelity in BCI applications (Fig. 9a). Piezoelectric films represent a valuable class of materials for BCI applications, offering the potential to harvest mechanical energy and potentially power self-sustaining BCI systems. Ren et al. [159] reported significant progress by developing freestanding (111)-oriented PbZr_0.52_Ti_0.48_O_3_ single-crystal thin films, which combine exceptional mechanical flexibility with notable piezoelectric performance. These films achieved an effective piezoelectric coefficient of approximately 585 pm V^−1^, nearly six times higher than the coefficient in their clamped state. The piezoelectric nanogenerators (PENGs) constructed from this material demonstrated an ultrahigh power density of about 63.5 mW cm^−3^, maintaining excellent mechanical resilience with strains exceeding 3.4% and stable output performance after 60,000 continuous bending cycles. This work not only sets a new benchmark for flexible PENG performance but also offers a promising self-powered solution for non-invasive BCIs (Fig. 9b). The development of ultrathin silver films (UTAFs) represents another significant advance in flexible optoelectronics. Ma et al. [160] developed 4.5 nm UTAFs, which exhibit 82% average visible transmittance, less than 60 ppm haze, and a sheet resistance of 7.6 Ω sq^−1^. These films exhibited excellent mechanical stability. The resistance increases by only 5% under static bending with a curvature radius of 3.5 mm, and by less than 7% after 100,000 dynamic folding cycles, outperforming commercial indium tin oxide electrodes. When applied in foldable alternating current electroluminescent devices, the UTAFs served as transparent bottom electrodes to maintain stable luminance, further confirming their mechanical and electrical stability. These characteristics suggest that UTAFs may support trends toward improved performance when implemented as transparent electrodes in flexible optoelectronic devices and intelligent hardware systems (Fig. 9c). Wang et al. [161] designed a taste interface that uses high-density, conformal tongue electrodes to capture electrical signals from the tongue. Combined with BCIs, the system enabled taste decoding of a reconstructed tongue without taste buds, achieving an accuracy of 97.8%. This approach provides a novel method for the clinical evaluation and treatment of patients with tongue cancer (Fig. 9d).

Additionally, Kim et al. [162] presented a support-free transfer method that facilitates the deposition of ultrathin single-crystalline SrRuO_3_ membranes onto flexible polyethylene terephthalate (PET) substrates, maintaining exceptional structural integrity, optical transparency, and electronic performance. These membranes, measuring approximately 2.5 by 2.5 square millimeters in clean surface area and with thicknesses as low as 15 nm, exhibited a high optical transmittance of about 60% in the visible spectrum. Their electrical resistivity at room temperature ranged between 10^–4^ and 10^–3^ Ω cm accompanied by robust ferromagnetic ordering that persists below 150 K. These SrRuO₃ membranes combine flexibility with functional performance, suggesting their possible value in developing flexible electronic and spintronic systems (Fig. 9e). Jin et al. [163] introduced a spontaneous bifacial capping strategy by incorporating 4-(methoxy)benzylamine hydrobromide (MeOBABr) into the perovskite precursor, markedly enhancing the mechanical stability and charge carrier transport properties of flexible perovskite solar cells. The work demonstrated that the nanoscale bifacial capping layers formed by MeOBABr effectively planarized grain boundary trenches, mitigated bending-induced stress, and improved charge extraction efficiency through surface defect passivation and band alignment optimization. The encapsulated target devices retained over 80% of their initial power conversion efficiency following 10,000 bending cycles at a curvature radius of 3 mm. Although primarily focused on photovoltaic applications, these findings provide valuable insights for material design in flexible electronics, such as long-term wearable BCIs (Fig. 9f). In summary, these materials demonstrate the diversity of approaches in developing flexible bioelectronics for non-invasive BCIs. Although they use different methodologies, they may exhibit favorable characteristics, including high conductivity and mechanical compliance that align with the requirements of wearable BCI design. Future integration of these materials into BCI systems could contribute to enhanced performance of non-invasive neural interfaces.

### One-Dimensional Nanowire Bioelectronic Systems for Signal Acquisition

Building on the flexibility and performance characteristics of conductive films, another emerging material strategy gaining attention in non-invasive BCI research involves the utilization of NWs [164]. NWs possess a unique one-dimensional (1D) topology, high aspect ratio, and tunable surface chemistry. These characteristics demonstrate potential for achieving the mechanical flexibility, electrochemical stability, and multimodal sensing capabilities required by BCIs [165]. Moreover, the structural features of NWs are believed to facilitate neural signal acquisition: their 1D morphology could promote directional charge transport, potentially reducing signal attenuation at heterogeneous biological interfaces.

**Fig. 10:**
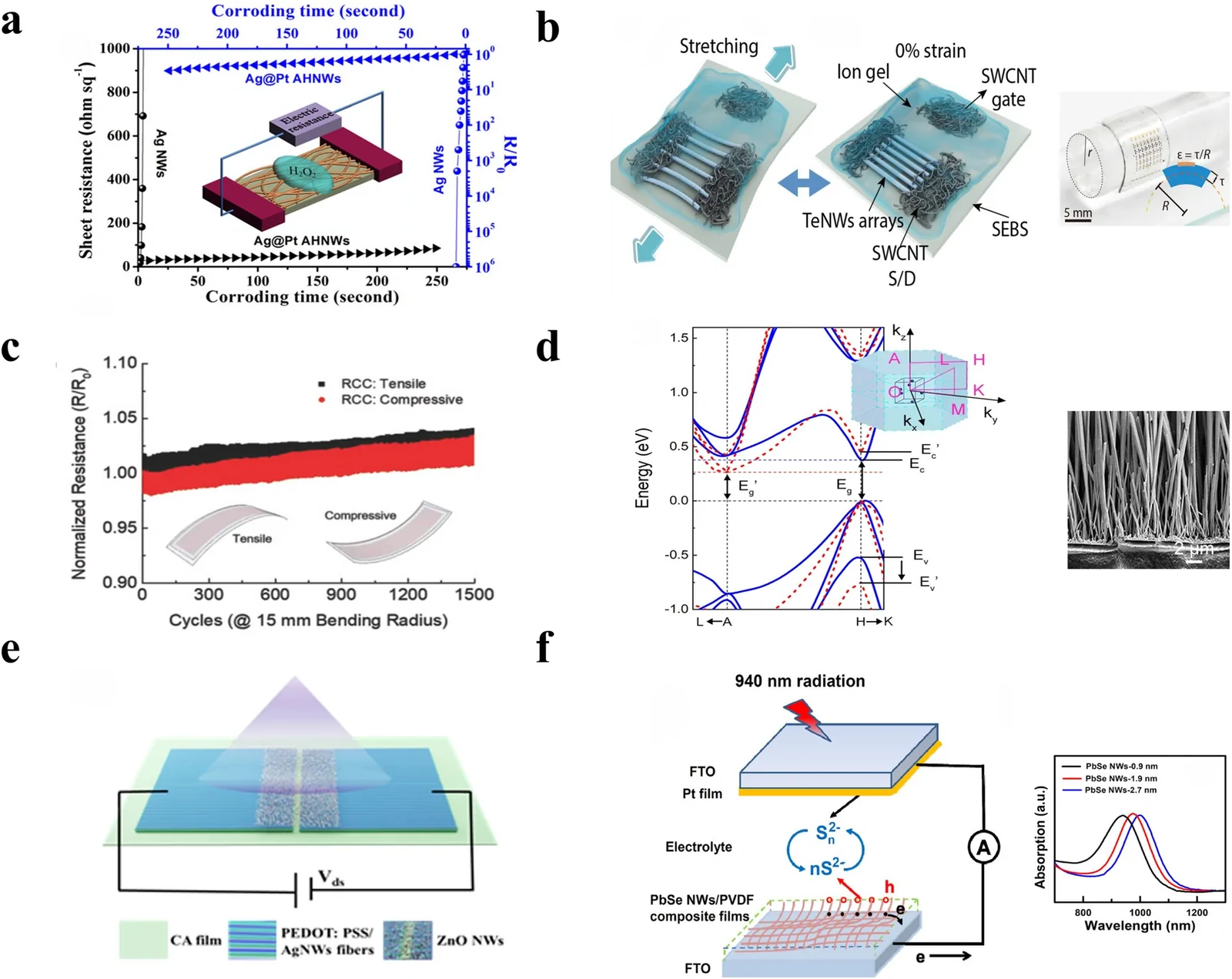
Schematic illustration of one-dimensional nanowire structures and properties. **a** Stability diagram of Ag@Pt AHNWs. Reproduced with permission [166]. Copyright 2018, American Chemical Society. **b** Left: Schematic of the structure of stretchable TeNW-TFTs before and after deformation. Right: Image of TeNW-TFTs under bending. Reproduced with permission [73]. Copyright 2024, The American Association for the Advancement of Science. **c** A cyclic bending test result of the flexible RCC electrode at a 15 mm bending radius. Reproduced with permission [167]. Copyright 2018, John Wiley and Sons. **d** Left: Band structure of Te illustrating its anisotropic electronic properties. Right: Side-view SEM image of vertically aligned TeNWs deposited on PI substrate. Reproduced with permission [168]. Copyright 2022, Springer Nature. **e** A Schematic of the fabricated fiber-based PD. Reproduced with permission [169]. Copyright 2025, Springer Nature. **f** Left: Schematic diagram of the device configuration of the representative self-powered PEC photodetectors based on PbSeNWs. Right: Normalized absorption spectra of PbSeNWs with different diameters. Reproduced with permission [170]. Copyright 2025, John Wiley and Sons

To overcome the instability of traditional silver nanowire electrodes, Zhou et al. [166] developed silver-platinum alloy-walled hollow nanowires (Ag@Pt AHNWs) through electrochemical and galvanic replacement processes. The silver-platinum alloy enhanced corrosion resistance, while the electrode maintained high optical transparency of 82% at a wavelength of 550 nm and a low sheet resistance of 28.73 Ω sq^−1^. The alloy structure exhibited excellent thermal stability, sustaining operation at 400 °C for 11 h, and demonstrated robust electrochemical stability in acidic environments, enabling integration into functional devices that require long-term reliability. This work provides a scalable approach for preparing highly stable metal nanomaterial electrodes, which is crucial for flexible optoelectronic devices operating in harsh environments (Fig. 10a). Zhao et al. [73] utilized a biomimetic lock-and-shear assembly strategy to fabricate wafer-scale aligned arrays of tellurium nanowires (TeNWs). The thin-film transistors exhibited hole mobility exceeding 100 cm^2^ V^−1^ s^−1^ and an on/off current ratio approaching 10^4^ on flexible PET substrates. Furthermore, devices fabricated on elastomeric substrates maintained stable electrical performance under uniaxial strains up to 40%, demonstrating excellent stretchability and mechanical robustness (Fig. 10b). Similarly, Kim et al. [167] demonstrated that copper nanowire electrodes encapsulated with polyurethane acrylate (PUA) exhibit significant environmental adaptability and mechanical stability. Specifically, after 1,500 bending cycles at a radius of 15 mm, the electrodes showed only approximately 3.5% increase in resistance under tensile testing and approximately 2.5% increase under compressive testing. Furthermore, under harsh conditions of 80 °C and 80% relative humidity, the PUA-coated electrodes maintained stable operation for up to 240 h, despite a twofold increase in resistance. This level of stability helps meet the need for reliable electrodes in applications that require long-term mechanical flexibility and environmental durability (Fig. 10c).

In addition to mechanical stability, NWs provide significant advantages in multimodal integration and functional expandability. Li et al. [168] leveraged the intrinsic anisotropy of tellurium NWs to create a bimodal tactile sensor (BTS) that achieves the decoupling of pressure and temperature difference signals. The material exhibited a carrier mobility of 1,000 cm^2^ V^−1^ s^−1^. When the mechanical pressure ranged from 0 to 5 kPa mechanical pressure, the sensor current increased from an initial 2.82–141.82 μA. Experimental results demonstrated that the BTS-based smart glove facilitated somatosensory feedback interaction between VR and the real world. By integrating sensor signals with deep learning techniques, successful stimulation recognition and neural reflex modeling of the rabbit sciatic nerve were achieved. Furthermore, the sensor exhibited excellent biocompatibility, offering broad application prospects in the biomedical field (Fig. 10d). Karagiorgis et al. [169] engineered a fully transparent and flexible photodetector by integrating ZnO NWs with electrospun PEDOT/PSS/Ag NW-based nanofibers on a biodegradable cellulose acetate (CA) substrate. The device demonstrated an ultraviolet responsivity of 1.10 × 10^6^ A W^−1^ under a 5 V bias and 0.5 μW cm^−2^ ultraviolet (UV) illumination intensity, alongside an optical transmittance of 70% at 550 nm wavelength. This combination of high performance and transparency was achieved while maintaining robust stability under dynamic UV exposure on flat and curved surfaces. Importantly, the CA substrate and PEDOT/PSS/Ag composite layers exhibited biodegradability within several months in buffer solutions, offering an eco-friendly solution to address electronic waste challenges. These results underscore the potential of this nanowire-based photodetector platform for next-generation wearable and transparent electronic applications (Fig. 10e). Li et al. [170] developed sub-1-nm PbSeNWs via a cation-exchange strategy in N, N-dimethylformamide (DMF) solvent, which exhibited a near-infrared (NIR) absorption peak centered at 940 nm. These self-powered photoelectrochemical photodetectors exhibited a responsivity of 113 mA W^−1^ and a detectivity of 4.65 × 10^11^ Jones without external bias. Embedded in flexible polyvinylidene fluoride (PVDF) composite films, these NWs combined superior carrier transport properties with mechanical flexibility. The NIR absorption at 940 nm aligned with the safety window for human tissue, providing a foundation for potential biomedical applications (Fig. 10f).

**Fig. 11:**
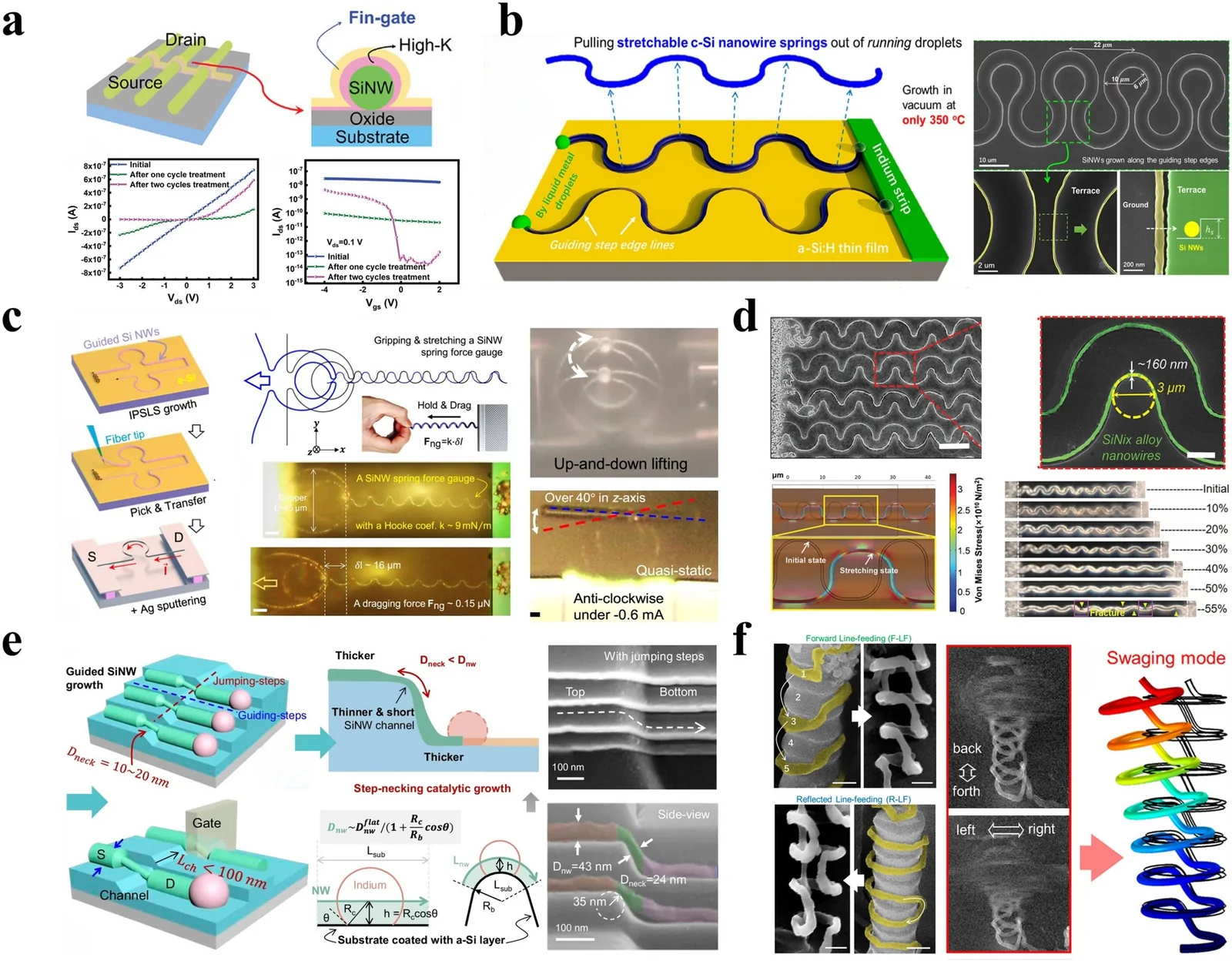
Schematic illustration of one-dimensional SiNW structures and properties. **a** Top: Fin-gate structure with aligned SiNW channels. Bottom: Ids-Vds output and transfer characteristics of SiNW TFTs. Reproduced with permission [175]. Copyright 2023, John Wiley and Sons. **b** Left: Pulling stretchable crystalline silicon (c-Si) nanowire springs out of running catalyst droplets. Right: SEM images of SiNWs. Reproduced with permission [176]. Copyright 2017, American Chemical Society. **c** Left: Fabrication of single-nanowire-morphed robotic grippers. Right: Elastic deformation, structural stability, and force gauging behavior of the nanowire gripper. Reproduced with permission [177]. Copyright 2023, Springer Nature. **d** Top: SEM images of SiNix nanospring arrays. Bottom: Microscopic images showing the geometric evolution of a SiNix-NS interconnection. Reproduced with permission [178]. Copyright 2021, John Wiley and Sons. **e** Left: Step-necking growth of ultrathin and short SiNW channels. Right: SEM image of SiNWs. Reproduced with permission [179]. Copyright 2025, Springer Nature. **f** Left: SEM images of SiNHs with forward- or reverse-line feeding growth symmetry. Right: Two distinct resonant frequencies corresponding to the swaging resonant modes of the SiNHs. Reproduced with permission [180]. Copyright 2020, American Chemical Society

Material innovation has contributed to recent progress in non-invasive BCIs, particularly through the development of nanomaterials that effectively combine electrical performance, mechanical flexibility, and biocompatibility. Silicon nanowires (SiNWs) exhibit compatibility with semiconductor processing, low-temperature fabrication capabilities, and controllable sub-100 nm architectures, suggesting their potential utility in neural interface applications [171–173]. These characteristics make SiNWs a potential platform for high-density flexible electrode arrays. Studies have highlighted their scalable fabrication and favorable electrical properties, which may support stable interfacing with biological tissues over extended periods [174]. For example, Song et al. [175] fabricated ultrathin SiNW arrays with uniform diameters of approximately 52 nm using an in-plane solid-liquid-solid (IPSLS) growth technique conducted below 350 °C. The SiNW thin-film transistors (TFTs) demonstrated a high transparency up to 90% and achieved an on/off current ratio greater than 10^6^. Notably, solution-based room-temperature passivation enhanced device stability. These advancements provide a foundation for high-fidelity neural signal recording in wearable electronics. Nevertheless, to facilitate widespread application in high-density neural interfaces, further improvements in the mechanical adaptability and environmental robustness of SiNW arrays are necessary (Fig. 11a). To address this, Xue et al. [176] demonstrated a method for deterministic structural programming of ultralong in-plane SiNWs. Utilizing the indium-droplet-guided IPSLS growth mechanism, they achieved the formation of highly stretchable c-Si springs or arbitrary two-dimensional patterns by directing catalyst droplets along predefined atomic step edges. In situ scanning electron microscopy (SEM), tensile testing, and electrical transport measurements revealed that the c-SiNWs maintained stable electrical connectivity under tensile strains exceeding 200%. This strategy represents a step toward the potential development of large-scale, wearable, and biocompliant electronic systems in future (Fig. 11b). Yan et al. [177] proposed an ultracompact Ω-shaped robotic gripper based on the deformation of a single nanowire and Lorentz force actuation. This gripper was capable of large-amplitude vibration and multidimensional operations, including grasping, lifting, and twisting. Additionally, it achieved precise payload release by overcoming van der Waals forces through high-frequency vibration. Paired grippers could collaboratively perform complex tasks such as microsphere transfer, demonstrating strong potential in high-sensitivity biosensing actuation. Looking ahead, such grippers could be explored as actuators in controlled neuromodulation platforms that interface with microscopic biological targets, where high precision and long-term biocompatibility would be key considerations (Fig. 11c).

To overcome limitations, Yuan et al. [178] fabricated an ordered array of ultrathin, highly conductive Si-Ni alloy nanowire springs (SiNix-NS) with an average diameter of approximately 160 nm. This innovation allows precise spatial control of SiNWs and the programmable design of elastic interconnect geometries. This approach is compatible with existing silicon-based thin-film technologies and addresses key limitations of conventional stretchable interconnect strategies. After nickel alloying, the electrical conductivity of the SiNixNWs increased by four orders of magnitude. When integrated onto flexible polydimethylsiloxane (PDMS) substrates, these nanowire springs exhibited excellent mechanical compliance, sustaining tensile strains exceeding 50% and maintaining stable electrical performance over 10,000 cycles at 15% strain. Owing to their ultracompact architecture and adaptable soft-interface compatibility, these nanowire springs demonstrate strong potential for low-damage, conformable integration in non-invasive BCIs (Fig. 11d). Concurrently, Wu et al. [179] used a step-guided necking growth method to fabricate ultrathin, short-necked SiNWs channels, which were designed for high-performance field-effect transistors (FETs). By tuning the size of indium (In) droplets and the height of the jumping steps, a thick-thin-thick short-channel structure could be formed. The necked region could be narrowed down to below 25 nm in diameter. These FETs exhibited a steep subthreshold swing of less than 70 mV dec^−1^ and an on/off current ratio exceeding 10^7^, significantly outperforming counterpart devices with uniform-diameter SiNW channels (Fig. 11e). Ma et al. [180] demonstrated a novel approach for the three-dimensional growth and integration of silicon nanohelices (SiNHs) on the sidewalls of bamboo-like cylindrical structures. In this method, periodic sidewall grooves were first formed via Bosch etching, and indium catalyst droplets were then employed to guide the helical growth of silicon along these grooves. By tuning growth parameters, the diameter, pitch, aspect ratio, and chiral/achiral symmetries of SiNHs could be precisely controlled. Furthermore, the SiNHs can be reliably released as individual units (Table 1). These SiNHs exhibit structural programmability and multifunctionality that may be advantageous for flexible bioelectronic systems, including non-invasive BCIs (Fig. 11f).

**Table 1:** Comparative Analysis of Key Parameters and Impacts of Nanomaterials in Non-Invasive BCIs

Materials	Conductivity	Young's Modulus	Stretchability	Stability	Direct Link to Non-Invasive BCI Performance	References
AgNWs	2.8 Ω sq^−1^	/	Up to 40%	∆Sheet Resistance ≈ ± 50% @ 100 cycles for 40%	Excellent signal quality; Middle mechanical durability; Middle monitoring density	[181]
AgNWs	3.2–3.6 Ω sq^−1^	/	/	0% increase in fractional resistance during fatigue testing up to 500,000 cycles	Excellent signal quality; Middle mechanical durability; Middle monitoring density	[182]
Nickel silicide NWs	2 × 10^6^ S m^−1^	170 GPa	Up to 50%	∆R/R_0_ < ± 1.7% @ 10, 000 cycles for 15%	Good signal quality; Middle mechanical durability; High monitoring density	[178]
SiNWs	Several uA under a bias voltage of ± 3 V	170 GPa	Up to 45%	stable ΔR/R0 @10, 000 cycles for 10%	Good signal quality; Middle mechanical durability; High monitoring density	[152]
PEDOT/PSS hydrogels	23.7 S m^−1^	8–374 kPa	Up to 100%	/	Good signal quality; Good mechanical durability; Middle monitoring density	[183]
d-Sorbitol-PEDOT/PSS hydrogels	> 1,000 S m^−1^	1.87 MPa	Up to 60%	Strain-insensitive resistance after the initial stretch	Good signal quality; Good mechanical durability; Middle monitoring density	[184]

### Zero-Dimensional Materials in BCIs: Innovation and Application

**Fig. 12:**
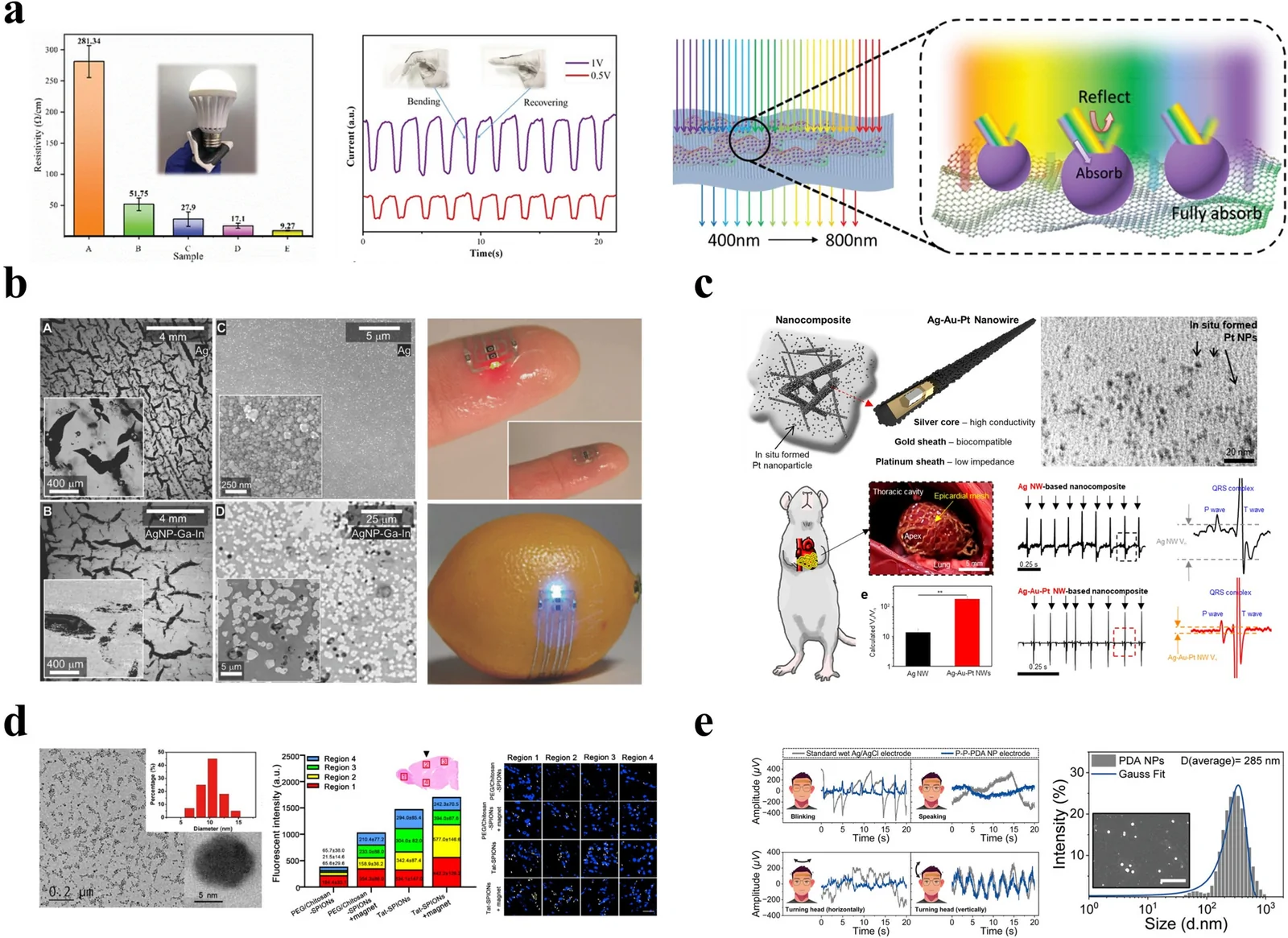
The application of zero-dimensional materials in BCIs. **a** Left: Resistivity of sensors with different graphene content at 2%, 4%, 6%, 8%, and 10%. Middle: Response tests of the film at different working voltages. Right: UV protection mechanism of the coating. Reproduced with permission [185]. Copyright 2021, John Wiley and Sons. **b** Left: The SEM images of printed AgNP and AgNP-Ga-In traces. Right: A circuit on a toy lemon and functioning as an electronic tattoo with an LED on a fingerprint. Reproduced with permission [186]. Copyright 2018, John Wiley and Sons. **c** Schematic of the Ag-Au-PtCore-Shell-Shell NWs, TEM images of the control nanocomposite, schematic illustration of the implantable electrode mounted on the rat's heart, and comparison of electrogram quality and magnified view of a single cardiac electrogram peak between Ag NW nanocomposite-based electrode and Ag-Au-Pt NW/Pt NP nanocomposite-based electrode. Reproduced with permission [187]. Copyright 2023, American Chemical Society. **d** Schematic of the nanoparticle synthesis process, quantification of the fluorescence intensity of RBITC, and representative confocal fluorescence images of brain slices of rats. Reproduced with permission [188]. Copyright 2022, Elsevier. **e** Motion artifacts comparison between the P-P-PDA NP electrode and the standard wet Ag/AgCl electrode, and characterization of PDA NPs. Reproduced with permission [189]. Copyright 2023, John Wiley and Sons

Zero-dimensional materials, such as nanocapsules and nanoparticles, often exhibit size-dependent properties different from those of their bulk counterparts, owing to their nanoscale dimensions. These distinctive characteristics are of significant importance for non-invasive BCIs. For non-invasive BCIs intended for long-term monitoring, the degradation of electrode materials significantly impacts signal acquisition quality. UV radiation is a critical factor in the aging process, particularly for certain organic flexible films. To address this issue, Zhou et al. [185] developed a multifunctional self-healing hybrid film composed of titanium dioxide nanocapsules, graphene, and multibranched polyurethane, exhibiting excellent mechanical flexibility and UV protection properties. Within an optimized graphene content range, the film maintained stable electrical properties, demonstrated a sensitive piezoresistive response, and possessed robust environmental adaptability. Notably, the film retained its conductivity and self-healing capability even after repeated mechanical damage. These attributes suggest potential applications in durable wearable human-machine interfaces (Fig. 12a). Furthermore, metal nanoparticles serve as excellent conductive fillers. Their integration with flexible substrates combines high electrical conductivity with mechanical flexibility. Such materials have been explored as electrode interfaces for non-invasive BCIs, where low impedance and stable skin contact are essential for reliable electrophysiological signal acquisition. Tavakoli et al. [186] introduced a room-temperature sintering technique using eutectic gallium-indium (EGaIn) to significantly enhance the electrical and mechanical properties of inkjet-printed silver nanoparticle (AgNP) traces for stretchable thin-film electronics. In this method, AgNPs with diameters around 100 nm were printed onto temporary tattoo paper and subsequently coated with a thin layer of liquid-phase EGaIn. This process induced the aggregation of AgNPs and filled microcracks, forming a continuous conductive Ag-Ga-In composite trace without requiring high-temperature treatment. The incorporation of EGaIn increased the electrical conductivity of the printed traces by six orders of magnitude, reaching 4.85 × 10^6^ S m^−1^, and improved the maximum failure strain from 4.5% to over 118%. The AgNPs played a critical role in forming conductive percolation networks and, upon interaction with EGaIn, contributed to the formation of heterogeneous Ag-In-Ga clusters that enhanced both conductivity and mechanical stretchability. The resulting circuits exhibited stable performance under strains up to 80%, low electromechanical coupling (gauge factor ≈1), and compatibility with hydrographic transfer to complex 3D surfaces and human skin. This approach facilitates the fabrication of highly deformable and robust electronic tattoos and stretchable sensors, offering a practical route toward skin-conformal and shape-adaptive electronics (Fig. 12b). Similarly, Sunwoo et al. [187] designed a stretchable low-impedance conductor based on Ag-Au-Pt core-shell-shell NWs and in situ synthesized platinum nanoparticles (Pt NPs) embedded in a styrene-ethylene-butadiene-styrene (SEBS) elastomer. The Ag-Au-Pt NWs feature a conductive Ag core, a biocompatible Au inner shell, and a low-impedance Pt outer shell with a highly embossed structure that enhances the effective surface area and charge transfer efficiency. The in situ formed Pt NPs, uniformly dispersed during composite fabrication, serve as critical conductive bridges between the NWs, reinforcing the percolation network. This synergistic structure yields a nanocomposite with high conductivity (~ 11,000 S cm^−1^), exceptional stretchability (~ 500%), low electrochemical impedance (166.5 Ω at 1 kHz), and significantly reduced cytotoxicity due to the effective suppression of Ag ion leaching. The incorporation of Pt NPs is essential for simultaneously enhancing electrical, mechanical, and electrochemical properties, supporting their use in high-performance wearable biosignal monitoring systems (Fig. 12c).

Beyond their role as conductive fillers, nanoparticles of certain materials exhibit unique enhancements in electromagnetic fields. For instance, iron oxide nanoparticles demonstrate superparamagnetism, which amplifies the electric field induced by the external field near the neuronal membrane, thereby exciting neuronal activity more effectively. Hong et al. [188] developed a non-invasive strategy for enhancing transcranial magnetic stimulation (TMS) using tailored superparamagnetic iron oxide nanoparticles (SPIONs) to improve functional recovery after ischemic stroke. They synthesized water-soluble Tat peptide-conjugated SPIONs (Tat-SPIONs) coated with chitosan and polyethylene glycol, which exhibit excellent colloidal stability and superparamagnetism. A combination of intranasal administration and external magnetic guidance facilitates the efficient, non-invasive delivery of these nanoparticles across the blood-brain barrier and into the brain parenchyma of rats. The delivered Tat-SPIONs significantly enhance the neurostimulatory effects of TMS, as evidenced by increased motor-evoked potential amplitudes, reduced motor thresholds, elevated c-fos expression, and marked improvements in motor-sensory and cognitive functions in a stroke model. Mechanistic investigations revealed that the enhancement was primarily mediated by a highly localized magnetoelectric effect from the plasma membrane-associated nanoparticles, which amplified the TMS-induced electric field to trigger neuronal activation. This platform demonstrates a viable pathway for medical translation of nanomaterial-enabled remote brain stimulation (Fig. 12d). What's more, certain nanoparticles, due to their hydrophilic/hydrophobic properties, hold significant potential for application in the interface engineering of non-invasive BCIs. This can be leveraged to ensure more intimate contact between the electrode and the skin. Han et al. [189] developed a flexible and self-adhesive hydrogel electrode for long-term wireless EEG recording and high-accuracy sustained attention evaluation. The hydrogel is constructed using biocompatible polyvinyl alcohol (PVA) and polyvinylpyrrolidone (PVP) via a ketalization reaction, resulting in a soft network with tissue-like modulus, high transparency, and excellent flexibility. To enhance functionality, polydopamine nanoparticles (PDA NPs) are incorporated into the hydrogel matrix through an oxidative degradation process, which converts opaque, micron-sized PDA aggregates into transparent, nanosized particles. The introduction of PDA NPs improves the hydrogel's self-adhesiveness, conductivity, and interfacial compatibility while maintaining high optical transparency and biocompatibility. The resulting multichannel electrode exhibits low interfacial impedance, high channel uniformity, low noise power, and robust performance under motion and sweating conditions. Moreover, the system demonstrated the ability to classify prefrontal EEG signals into seven levels of sustained attention with 91.5% accuracy using a linear support vector machine (LSVM) classifier. This nanoarchitectonics strategy highlights the critical role of PDA NPs in enabling high-performance, multifunctional hydrogel electrodes for personalized health monitoring and cognitive state assessment (Fig. 12e).

### Wearable Flexible Devices: Design Principles and Functional Integration

#### Design Principles

**Fig. 13:**
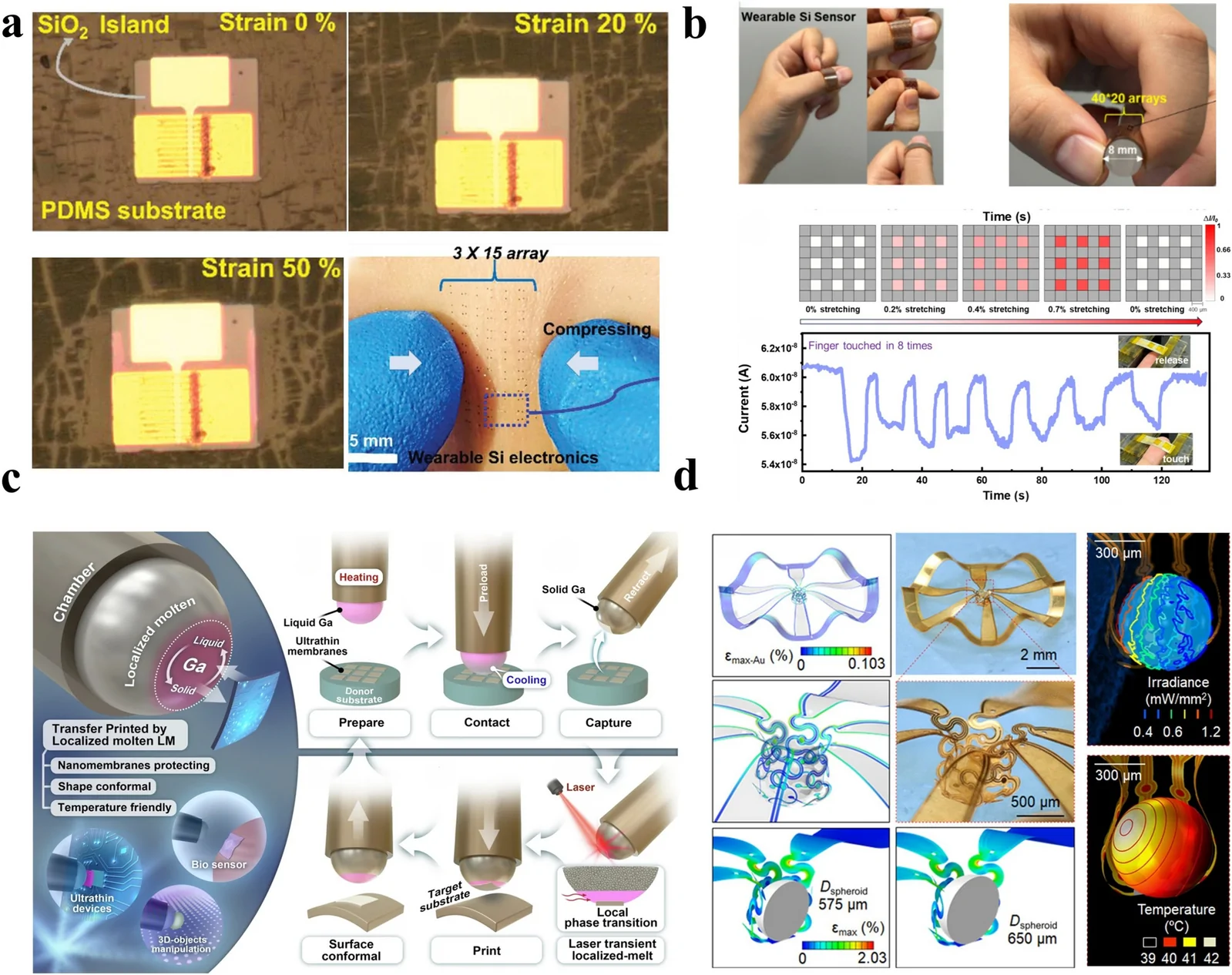
Wearable flexible devices: design principles. **a** Magnified photo of the hard island region at various strains on the PDMS film and the array attached to human skin. Reproduced with permission [192]. Copyright 2022, John Wiley and Sons. **b** Top: Optical image of a flexible SiNW strain sensor array attached to a human finger. Bottom: Normalized current maps of a sensor matrix under varied strains and its real-time response. Reproduced with permission [193]. Copyright 2025, American Chemical Society. **c** Schematic illustration of the pickup and printing process of PLMT. Reproduced with permission [194]. Copyright 2024, Springer Nature. **d** Left: 3D MMF with serpentine wires. Right: Simulated 3D distributions of light and temperature on neural spheroids. Reproduced with permission [195]. Copyright 2021, The American Association for the Advancement of Science

As the development of non-invasive BCIs progresses, the integration of wearable flexible devices has become a key area of focus. These devices aim to bridge the gap between advanced bioelectronic systems and user-friendly, comfortable applications [154]. To achieve this, the design of flexible electronics must strike a delicate balance between mechanical flexibility, biocompatibility, and signal fidelity-ensuring that devices can comfortably interface with the human body while maintaining high performance over extended periods of use [190, 191]. The rapid evolution of flexible bioelectronics is driving advances in the landscape of wearable BCIs, enabling opportunities for real-time monitoring and seamless interaction with dynamic biological systems. Silicon is the most mature and reliable semiconductor material in current manufacturing processes, and is widely used in electronics and sensing. However, integrating silicon with flexible substrates to extend its applications into flexible electronics poses a significant challenge. As the fundamental building block of modern integrated circuits, transistors hold great potential in non-invasive BCIs due to their gating and current amplification capabilities. Yet, limited by the intrinsic brittleness of silicon, transistors on flexible films often suffer structural damage due to modulus mismatch. To address this, Song et al. [192] directly integrated transistors onto a flexible film using a strategy based on rigid-island protection. This design maintained functional integrity under 50% tensile strain and supported 1,000 mechanical cycles at 20% strain, proving the robustness and durability of these devices. These SiNWs FETs exhibited a hole mobility of 70 cm^2^ V^−1^ s^−1^, an on/off current ratio exceeding 10^5^, and subthreshold swing values ranging from 134 to 277 millivolts per decade. Notably, the devices demonstrated stable electrical operation over a period of 270 days under ambient conditions without any encapsulation or passivation layers. Optical and scanning electron microscopy images confirmed conformal skin adhesion during deformation, while finite element analysis revealed that the stress-optimized island layout effectively minimized interfacial strain, supporting long-term mechanical stability (Fig. 13a). Furthermore, integrating nanomaterials with flexible films to combine mechanical flexibility and electrical conductivity is a widely adopted strategy. This is often achieved by directly transferring nanomaterials onto flexible films using a post-transfer technique. However, this method faces challenges such as the difficulties in precise integration, potential device non-uniformity, and low pixel density. Consequently, the challenge of fabricating high-density, precisely integrated chips, akin to those produced by top-down etching processes like photolithography, presents a significant hurdle for developing higher-density, higher-performance non-invasive BCIs. To address this, Song et al. [193] directly grew elastically deformable SiNWs on a flexible polyimide film and integrated them into high-performance strain sensors. This architecture combines structural elasticity and conformability, offering a robust platform for continuous biomechanical monitoring in soft-interface neuroelectronics (Fig. 13b).

Beyond the direct fabrication and integration of electronic devices on flexible films, transfer printing also serves as a rapid and effective strategy. However, when transferring exceptionally thin electronic devices or electrodes onto flexible films or biological interfaces, issues such as structural fractures due to stress concentration or delamination caused by poor interfacial adhesion may arise. To address this challenge, Shi et al. [194] developed a phase-transition-enabled gallium stamp that facilitates damage-free, three-dimensional transfer printing of ultrathin functional membranes. This method achieved seamless integration onto complex curved surfaces, including fingernails and contact lenses, with an average displacement across the array of 21.9 µm. By harnessing gallium’s reversible solid-liquid-phase transition, the stamp applied an exceptionally gentle preload of 0.0053 MPa while achieving robust adhesion up to 0.15 MPa. Notably, the approach reduced interfacial shear strain by four orders of magnitude compared to conventional PDMS stamps, significantly mitigating mechanical mismatch and delamination risk. These capabilities highlight the technology's potential for integration into non-invasive EEG systems, where mechanical stretchability and long-term skin conformance are essential for stable signal acquisition over irregular scalp geometries (Fig. 13c). Structural engineering, as one of the most widely adopted strategies in flexible electronics, also holds significant potential for non-invasive BCIs. It can partially compensate for the limitations of materials' intrinsic properties. In non-invasive BCIs, modulus matching at the biointerface is crucial. Matched Young's modulus tends toward achieving conformal contact with the epidermis, suggesting the potential to reduce contact impedance, improve the SNR, and mitigate motion-induced artifacts. Such mechanical compatibility could also contribute to improved comfort during extended wear. For instance, designs such as wavy, serpentine, and fractal structures facilitate better modulus matching with the biointerface and potentially enhance the mechanical stability of non-invasive BCIs under dynamic conditions. In scenarios requiring microscale 3D neural monitoring, traditional thin-film electrodes often fall short. Therefore, structurally designing electrodes to achieve conformational matching with the target measurement sites is of great importance for the advancing field of BCI technology. Park et al. [195] proposed a three-dimensional multifunctional neural interface platform designed for cortical spheroids and engineered neural assembloids. This platform leverages mechanically guided assembly techniques to transform planar structures into 3D architectures, enabling multifunctional interactions with neural spheroids. Utilizing ultrathin polyimide substrates and microscale gold electrodes, the system achieved high-fidelity detection of action potentials, with an average signal duration of 0.5 ms and peak-to-peak amplitudes reaching 15 microvolts. The platform exhibits favorable mechanical compliance, with relatively low bending stiffness that facilitates conformal contact with soft biological tissues. Its elastic interconnects remain functional under small applied strains, enabling stable and reasonably conformal integration near the cortical surface. This research also demonstrated the potential platform's multifunctionality across electrical, optical, thermal, and chemical modalities, with experimental validation of its capabilities in monitoring neural activity and investigating neural injury and recovery (Fig. 13d).

#### Functional Integration

**Fig. 14:**
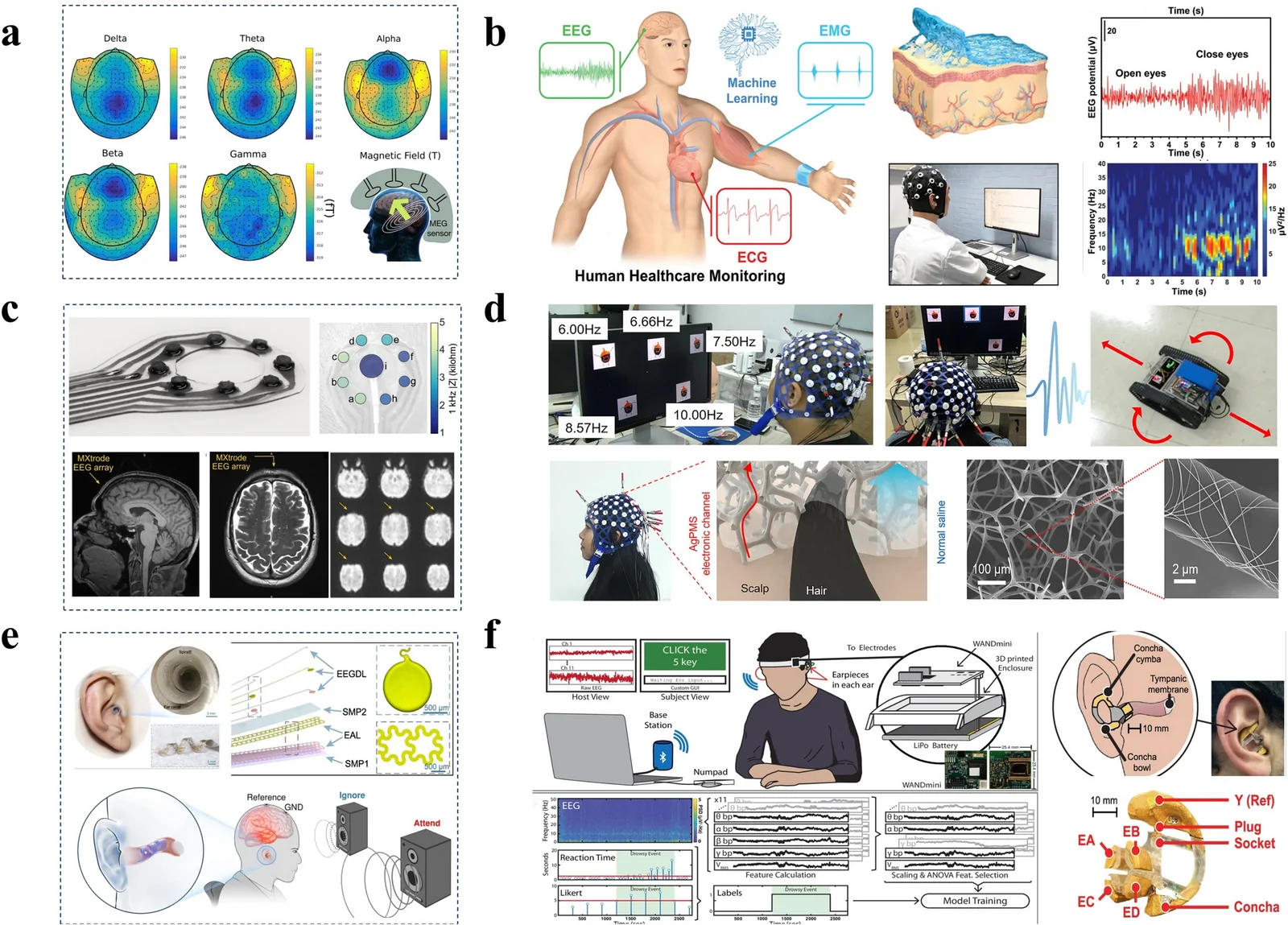
Wearable flexible devices: functional integration. **a** Compatibility of TTE with MEG. Reproduced with permission [196]. Copyright 2020, Springer Nature. **b** Left: Schematic diagram of EEG signal monitoring. Right: EEG signals recorded with corresponding spectrogram in the eyes-closed state. Reproduced with permission [197]. Copyright 2024, John Wiley and Sons. **c** Top: Impedance map at 1 kHz for all electrodes on the subject's head. Bottom: a 3-T clinical scanner. Reproduced with permission [198]. Copyright 2021, The American Association for the Advancement of Science. **d** Left: Contact analysis and EEG signal recording. Right: The SEM images of the AgPMS. Reproduced with permission [199]. Copyright 2019, American Chemical Society. **e** Top: Design of in-ear bioelectronics. Bottom: Schematic diagram of the cocktail effect experiment based on SpiralE. Reproduced with permission [9]. Copyright 2023, Springer Nature. **f** Left: Experimental setup for EEG acquisition and drowsiness labeling. Right: Earpiece assembly and fit. Reproduced with permission [28]. Copyright 2024, Springer Nature

Building on these advances in flexible electronics, the next step is applying them specifically to non-invasive BCIs. With the advancement of non-invasive BCI technology, EEG monitoring is no longer confined to short-term, static observations, as long-term dynamic monitoring is garnering increasing attention. However, when BCIs monitor moving subjects, new challenges are posed to the mechanical durability of electrodes. Prolonged dynamic operation could introduce structural defects from mechanical fatigue within the electrodes, leading to a degradation in their EEG signal detection capability. Consequently, materials capable of self-recovery hold significant potential for enabling stable, long-term monitoring with non-invasive BCIs. Ferrari et al. [196] fabricated ultrathin conductive polymer tattoo electrodes (TTEs) using inkjet printing technology and validated their performance in clinical EEG as well as their compatibility with MEG. TTEs were able to detect alpha waves with significantly higher signal amplitude around 20 Hz compared to Ag/AgCl electrodes, demonstrating greater sensitivity. Moreover, during auditory evoked potential recordings, TTEs exhibited a superior SNR (4.07 vs. 3.36) relative to conventional electrodes. TTEs also conformed closely to the skin surface and were less affected by hair, making them well-suited for long-term monitoring. Therefore, this technology holds promise for potential applications in multimodal brain monitoring and diagnostics (Fig. 14a). Simultaneously, relative motion between a non-invasive BCI and the body interface can introduce artifacts during measurement, which severely compromise the quality of the acquired signals and complicate the extraction of target neural information. Therefore, maintaining stable contact between the non-invasive BCIs and the target interface is paramount. Bioadhesive materials, capable of conforming intimately to biological surfaces, offer low interfacial impedance and favorable mechanical flexibility. This presents an effective strategy for achieving high SNR and stable monitoring with non-invasive BCIs. Wang et al. [197] designed an epidermal sensor based on bioadhesive MXene hydrogel, in which a dynamic cross-linked network provided excellent conductivity retention under 200% tensile strain and rapid mechanical self-healing. These properties contribute to maintaining stable performance in scenarios involving complex skin deformations. By synergistically integrating conductive MXene nanosheets with bioadhesive functional groups, the hydrogel interface achieved tunable adhesion strength ranging from 10.17 to 38.75 kPa, maintaining stable electrode-skin impedance during motion and supporting high-fidelity EEG signal acquisition. The three-dimensional porous architecture offered not only ultraviolet shielding but also antibacterial properties, with inhibition rates of 89.61% and 93.15% against Escherichia coli and Staphylococcus aureus, respectively, ensuring long-term wearability. Integrated with a machine learning algorithm, the system achieved an accuracy of 98.1% in EMG-based sign language recognition. This multifunctional platform holds great potential for advancing next-generation wearable electronics and machine learning-assisted human-machine interaction (Fig. 14b). The electrodes used in non-invasive BCIs can be simply classified according to material properties into three types: wet, semi-dry, and dry. Wet electrodes have been extensively applied in clinical practice. A key advantage lies in their use of an electrolyte medium that fills interfacial gaps on the skin, enabling effective contact. This mechanism facilitates low interface impedance and allows for stable signal acquisition with a high SNR. However, the intrinsic properties of wet electrodes render them unsuitable for long-term monitoring. On the one hand, the conductive gel applied at the biointerface to facilitate contact can dry out over extended periods, leading to a significant degradation of signal acquisition capability. On the other hand, wet electrodes can cause subject discomfort, and prolonged skin contact with the moisture may induce irritation or inflammation. Hence, dry electrodes are regarded as well-suited for long-term non-invasive BCI monitoring applications. Driscoll et al. [198] developed MXtrodes, a gel-free, MXene-based bioelectronic platform for high-resolution electrophysiological recordings in neural and neuromuscular systems. In non-invasive EEG applications, 3D pillar-shaped MXtrodes achieved precise acquisition of alpha rhythms and motor-related mu suppression signals, with signal quality comparable to clinical-grade gelled electrodes. The devices exhibited excellent mechanical compliance, scalable laser-based fabrication, and strong compatibility with MRI and CT imaging, collectively contributing to the mitigation of several key challenges in wearable neurotechnology. This work establishes MXene composites as a key materials platform capable of advancing adaptive, multimodal bioelectronic systems that bridge clinical-grade and consumer-grade BCIs (Fig. 14c). Among the numerous parameters influencing BCIs, hair and sweat are notably significant sources of interference. Hair could impede intimate contact between the electrode and the scalp, increasing contact impedance, reducing the SNR, and amplifying motion-induced artifacts. Sweat, due to its ionic content, alters the direct interfacial impedance, leading to DC drift and slow-wave artifacts. To mitigate the interference from hair, strategies can be devised from the perspectives of electrode structure and material. For instance, replacing planar film electrodes with spike-shaped electrodes can effectively bypass hair and establish stable contact directly with the scalp. Alternatively, employing porous materials like a sponge as the electrode substrate allows it to compress around hair follicles under application pressure, maintaining consistent contact with the scalp. Lin et al. [199] developed a flexible, gel-free silver nanowire-based sponge electrode (AgPMS), which exhibits high conductivity and excellent mechanical and chemical stability, allowing for effective contact with the skin, bypassing hair. In SSVEP experiments, AgPMS achieved a classification accuracy of 86% on hairless skin and 82% on hairy skin, a performance closely approaching the 88% accuracy of standard gel electrodes. These results demonstrated that this electrode can significantly improve the performance of gel-free, non-invasive BCIs, providing a superior option for EEG recordings in applications such as assistive devices for individuals with disabilities and mental state monitoring. It holds significant clinical and practical value, particularly for real-world, hair-covered non-invasive BCI applications (Fig. 14d).

Emerging ear-centric neural interfaces capitalize on the ear's unique anatomical advantages, such as hairless regions for stable electrode contact, inherent mechanical stability that helps mitigate motion artifacts, and compact form factors compatible with wear. These combined features indicate long-term neural monitoring. Wang et al. [9] developed SpiralE, an in-ear bioelectronic platform that conforms to the shape of the ear canal. It autonomously expands and spirals along the ear canal under electrothermal actuation, ensuring optimal contact with the ear canal for reliable EEG sensing. This makes it suitable for the construction of both visual and auditory BCIs. In a 9-target SSVEP BCI classification, offline accuracy reached 95%, and online accuracy in a calibration-free 40-target SSVEP speller experiment was 75%. For auditory attention decoding in cocktail party scenarios, natural speech classification accuracy achieved 84% (Fig. 14e). Complementing this, Kaveh et al. [28] developed a wireless dry-electrode in-ear EEG monitoring system for drowsiness detection. During 35 h of drowsiness monitoring across nine participants, the support vector machine model achieved an average drowsiness event detection accuracy of 93.2% for old users and 93.3% for new users (Fig. 14f). These results validate the feasibility of wireless dry user-generic earpieces for drowsiness classification and lay the groundwork for population-trained classification in future electrophysiological applications.

### Integration of Flexible Electronics and Deep Learning for Advanced Non-Invasive BCIs

Recent performance gains in non-invasive BCIs have been enabled by the co-design of flexible bioelectronics and deep learning-based decoding algorithms. This synergistic paradigm establishes a positive feedback loop: advanced materials and interface engineering provide a high-fidelity signal foundation for algorithms, while intelligent algorithms, in turn, compensate for the inherent physical limitations of hardware and guide the design direction of future hardware, collectively pushing the boundaries of system performance.

Building upon material innovations, Yang et al. [200] developed a biosensor based on an adhesive-hydrophobic bilayer hydrogel (AHBH), which was integrated into a portable head-mounted device for high-fidelity EEG-based emotion classification. Serving as the core interface, the AHBH material leverages a mussel-barnacle-inspired bioadhesion mechanism combined with a hydrophobic surface reorganization strategy. This design achieves a dry-state adhesion strength of 59.7 N m⁻^1^ and a water contact angle of 133.87°, effectively blocking sweat penetration, suppressing motion artifacts, and maintaining an adhesion strength of 40.8 N m⁻^1^ after 20 peeling cycles, thereby demonstrating excellent skin adhesion stability. The material's low elastic modulus (≈6.9 kPa) and high stretchability (≈690%) ensure conformal contact with the skin during deformation, significantly reducing contact impedance compared to commercial Ag/AgCl electrodes and Ecoflex-ECC electrodes, along with minimizing signal drift and noise. Consequently, the AHBH-ECC electrode exhibits exceptional electrical stability in dynamic environments such as vibration and sweating conditions, with its noise RMS value measuring only 9.5 ± 1.6 μV. This performance is substantially superior to that of Ag/AgCl electrodes (46.3 ± 4.6 μV) and Ecoflex-ECC electrodes (93.0 ± 7.8 μV), indicating enhanced resistance to motion artifacts. The high-quality raw signals facilitate the implementation of more complex algorithms: the study introduced a combination of differential entropy and power spectral density features to enhance emotional state differentiation and employed a domain adaptation neural network to compensate for residual hardware noise and cross-subject variability. This approach ultimately increased the average emotion classification accuracy from approximately 85% with traditional neural networks to 90%. An important direction for future research is to quantify the marginal improvement in final classification accuracy attributable to the hydrogel interface through direct comparison with conventional electrodes (Fig. 14a).

Extending the co-design concept to system-level integration, Mahmood et al. [201] developed a wireless soft bioelectronic BCI platform that integrates dry microneedle electrodes, stretchable interconnects, and a VR headset. The interconnect structure demonstrates negligible resistance change after 100 cycles at 50% strain, ensuring signal stability during dynamic movement. At the signal level, this architecture effectively suppresses motion artifacts and electrostatic interference, thereby enhancing both the SNR and recognition performance of SSVEP. For feature extraction and algorithm implementation, the study introduced a split-eye asynchronous stimulation (SEAS) paradigm that expands the number of distinguishable frequency combinations to 32, subsequently employing a spatial convolutional neural network (Spatial-CNN) to achieve high-resolution classification within a short 0.8 s time window. Under a configuration of 33-class stimuli and 4-channel acquisition, the system achieved a classification accuracy of 78.93% ± 2.36% and an ITR of 243.6 ± 12.5 bits min^−1^, significantly outperforming the traditional Ag/AgCl electrode system, which achieved an accuracy of 74.72% ± 3.03% and an ITR of 222.4 ± 15.0 bits min^−1^. The system was demonstrated in real-time applications, including VR text spelling and virtual environment navigation. None of the nine subjects reported discomfort during one hour of continuous use, indicating satisfactory wearability and user experience. Future studies building on this work could expand participant diversity to include individuals with motor disabilities and evaluate long-term wearing comfort through extended-use trials (Fig. 14b).

Further advancing the hardware-algorithm closed-loop integration, the same research team reported the SKINTRONICS system [10]. This system utilizes flexible circuits and dry electrodes composed of ultrathin aerosol-jet printed skin electrodes and flexible conductive polymer, with a mechanical modulus highly matched to scalp tissue to achieve conformal contact. This configuration effectively reduces electrode-skin contact impedance to below 20 kΩ, thereby significantly suppressing signal artifacts and electromagnetic interference caused by relative interfacial motion. The system enables high-quality, stable EEG signal acquisition, providing reliable input for subsequent deep neural network processing. Quantitative experiments demonstrated that the system achieves an SNR of 46.6 ± 2.16 dB in SSVEP detection, performing significantly better than traditional gel electrode systems (16.94 ± 4.60 dB) and existing portable wireless systems (28.89 ± 2.28 dB). Concurrent reliability tests confirm excellent long-term stability: the flexible circuit exhibits resistance changes of less than 0.09 Ω after repeated 180° bending around a radius as small as 1.3 mm, while maintaining stable wireless signal strength within a 15-m range. Additionally, the elastomer hairy electrode shows less than 10% change in contact impedance after 1,000 compression cycles. On the algorithmic side, a grid-search optimized dual-layer CNN enables automatic extraction of robust features directly from two-channel time-domain SSVEP signals, effectively overcoming the performance limitations of traditional methods with limited channels. The cross-subject generalization capability of the dual-layer CNN was validated through a six-fold cross-subject validation, where models trained on 5 subjects were tested on an independent subject. Benefiting from this hardware-software co-optimization, the system achieved an offline classification accuracy of 94.54% ± 0.9% and an ITR of 122.1 ± 3.53 bits min^−1^ using only two EEG channels. The system successfully translates decoded features into control commands, enabling real-time, precise control of an electric wheelchair, a wireless vehicle, and demonstration software (Fig. 14c).

**Fig. 15:**
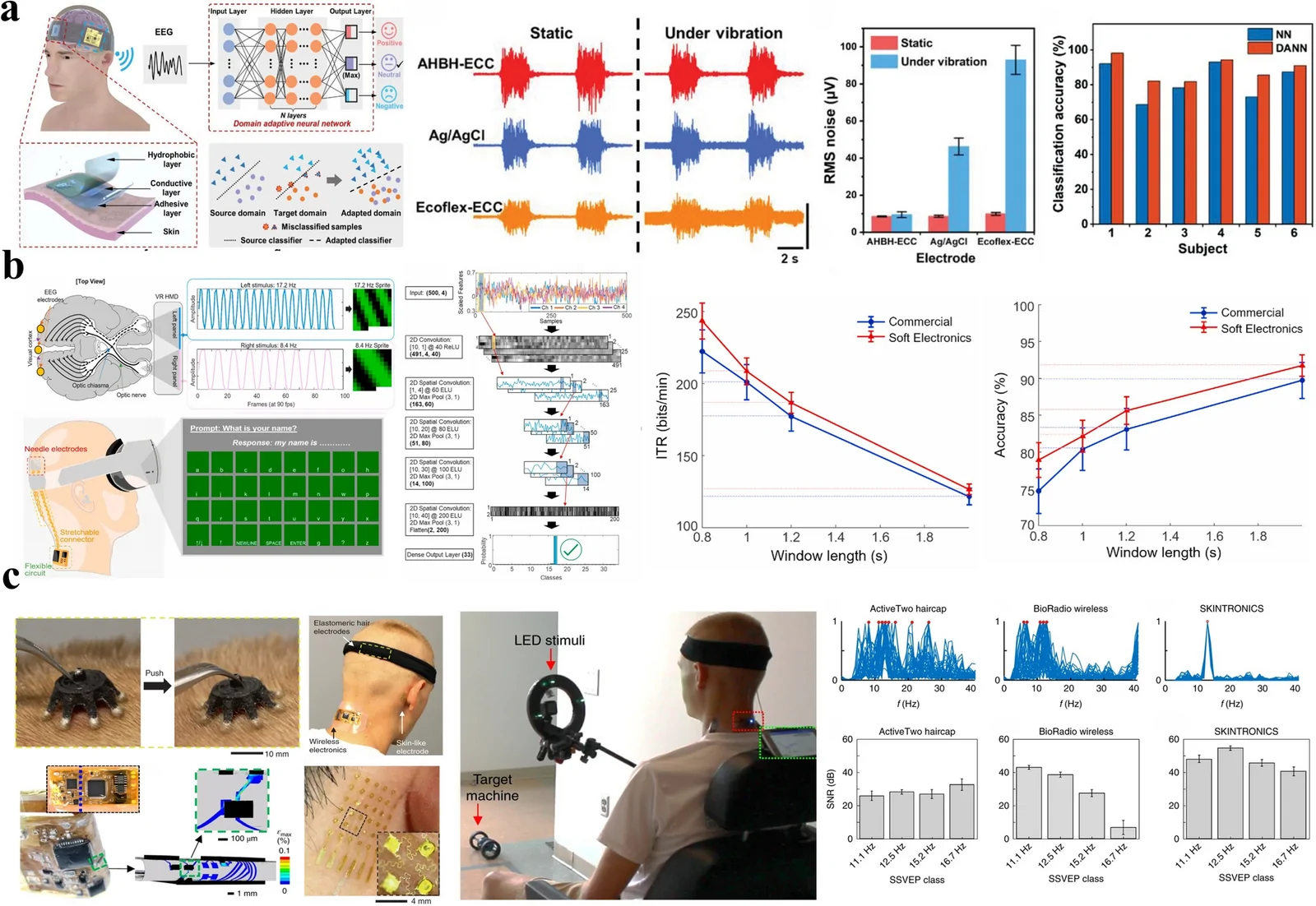
Integration of flexible electronics and deep learning for advanced non-invasive BCIs. **a** Schematic illustration and performance of the AHBH skin-interfaced biosensor for human emotion classification. Reproduced with permission [200]. Copyright 2022, John Wiley and Sons. **b** Schematic of VR-enabled portable BCIs. Reproduced with permission [201]. Copyright 2022, Elsevier. **c** Schematic of the system architecture featuring fully portable and wireless scalp electronics. Reproduced with permission [10]. Copyright 2019, Springer Nature

In summary, these innovative approaches collectively outline a clear trajectory for synergistic development in non-invasive BCIs. Advancements in flexible electrodes establish the hardware foundation by optimizing interfacial contact and suppressing motion artifacts, thereby consistently providing more stable, high-SNR raw signals that supply high-quality, high-fidelity data for subsequent decoding. Building upon this foundation, advanced algorithms such as deep learning leverage their powerful feature extraction capabilities to not only achieve significant improvements in decoding performance compared to traditional methods but also actively compensate for inherent hardware limitations, including residual noise and cross-subject variability. This synergistic paradigm, embodying the principle of hardware establishing the foundation and algorithms driving advancement, creates a positive feedback loop that collectively expands the performance boundaries of non-invasive BCIs (Fig. 15).

Looking ahead, advancing beyond current performance limitations requires the development of specialized hardware designed for new computing paradigms. The traditional von Neumann architecture encounters energy efficiency bottlenecks when processing neural signals, whereas neuromorphic computing approaches, such as those utilizing memristor-based in-memory computing chips combined with event-driven spiking neural networks, offer a promising solution for ultra-low-power edge computing [151]. This calls for hardware-software co-design, involving cross-level optimization from materials and devices to algorithms. Such co-design ensures deep integration of hardware physical characteristics and algorithmic computational requirements from the initial design stages, while avoiding latency and privacy risks associated with cloud transmission. Ultimately, translating these laboratory innovations into reliable user assistive tools demands rigorous validation in real-world environments. The principal future challenge involves systematically evaluating the decoding robustness, long-term wear comfort, and biocompatibility of these co-designed systems across diverse populations during continuous multiday monitoring, while simultaneously addressing accompanying system-level engineering issues to complete the crucial transition from technological prototype to practical product.

### Systems-Engineering for Non-Invasive BCIs

In multisubject collaborative non-invasive BCI systems, precise clock management poses a critical challenge for ensuring data timeliness and comparability. The core issue stems from inherent discrepancies in the internal clock sources of independent acquisition devices. Due to manufacturing tolerances of crystal oscillators and of environmental influences such as temperature and voltage fluctuations, these clocks exhibit varying degrees of drift and offset. Consequently, temporal misalignment gradually develops across devices, making it difficult to reliably synchronize neural responses to the same stimulus from different subjects. This synchronization problem is especially pronounced in medical applications where communication firmware in clinical-grade devices is often closed and restricted by regulatory requirements, preventing access to precise timestamps at the hardware level; timestamps can only be recorded at the application layer, introducing substantial indeterministic and variable software and communication delays, which significantly increase synchronization complexity. A representative collaborative solution combines dedicated hardware modules with synchronization software protocols. A synchronization hub is deployed within the network alongside multiple distributed synchronization plugins. Through periodic reference message broadcasting, timestamp exchange among nodes, and dynamic reference node election, each node applies linear regression to continuously estimate and compensate for its own clock offset relative to the reference node. This allows the system to maintain network-wide temporal consistency even under unstable communication latency conditions [202]. When hardware-level alignment is infeasible, software frameworks, such as the lab streaming layer, could achieve network-based timestamp calibration [87, 88], while dynamic time warping and event resampling could correct residual drift during post-processing. Future systems will require synchronization mechanisms that combine millisecond-level real-time performance, cross-device stability, and cross-session reproducibility to support high-precision applications such as clinical diagnostics, collaborative BCIs, and large-scale hyperscanning studies.

In non-invasive BCIs such as EEG, power consumption and thermal management represent major challenges [203]. Although such systems do not require surgical implantation, their power dissipation may still lead to localized temperature increases, affecting user comfort and long-term safety [204]. Because EEG electrodes are in direct contact with the scalp—where blood perfusion is relatively low and thermal conductivity is poor—heat generated by signal acquisition, amplification, or wireless transmission modules is difficult to dissipate efficiently. This may result in local temperature elevation that approaches or exceeds biological safety thresholds. Furthermore, non-invasive devices are typically portable and battery-powered [63]. Therefore, power efficiency not only affects battery life but also directly impacts wearability and biocompatibility. As such, low-power circuit design, efficient thermal dissipation structures, and proper material selection must be thoroughly considered during the hardware design stage. Thermal simulation tools should be used to evaluate temperature distribution under various physiological and environmental conditions to ensure that devices meet functional requirements while complying with thermal safety standards and enhancing user experience. Integrated design is considered a key strategy for improving power efficiency. For example, microchip-based integration of EEG and fNIRS sensors on a single platform reduces device size and weight, lowers power consumption, and enhances signal quality and synchronization.

Electromagnetic compatibility (EMC) presents a significant challenge throughout the signal acquisition, transmission, and processing pipeline of non-invasive BCIs. The issue arises from a fundamental contradiction: BCIs are designed to detect extremely weak neural electrophysiological signals (e.g., EEG) on the order of microvolts, while operating in environments that inevitably contain strong internal and external electromagnetic interference. This interference includes power line radiation at 50/60 Hz and its harmonics, as well as noise from consumer electronics such as Wi-Fi and Bluetooth devices. Additionally, physiological artifacts generated by the user—such as EOG or EMG—can have amplitudes much larger than EEG signals. These disturbances can be introduced into the system through electrodes or leads acting as "antennas", which deteriorate signal quality, distort data, and may result in misclassification or system malfunction. This presents direct risks to the reliability and safety of BCI systems in applications such as monitoring, diagnosis, or neuro-assistive tasks. To address this complex issue and ensure stable system performance in real electromagnetic environments, a comprehensive engineering strategy is required. This strategy must integrate hardware design, signal processing, and preliminary evaluation [205]. On the hardware side, shielded cables, driven right-leg circuits, and other active shielding techniques are used to minimize electromagnetic penetration, while amplifiers with high input impedance and high common-mode rejection ratios are employed to enhance the detection of weak differential signals. On the algorithmic side, residual noise persists despite hardware optimization, necessitating advanced signal processing techniques such as blind source separation, adaptive filtering, and deep learning models to extract meaningful neural data from contaminated signals [136]. During the device development phase, combining EMC analysis with numerical simulations of virtual human models has become an essential step in verifying compatibility and safety in realistic tissue environments. Collectively, these layered EMC strategies are essential for improving system SNR and facilitating the transition of non-invasive BCIs from controlled laboratory settings to practical real-world applications.

In non-invasive BCI systems, firmware update and rollback mechanisms are critical for ensuring long-term safety and reliability. However, these mechanisms remain underexplored. Given that BCI devices operate in close and sustained contact with the human body, firmware updates affect not only algorithm performance but also user safety and ethical accountability. Without integrity verification and rollback pathways, faulty updates may disrupt signal acquisition, render devices inoperative, or introduce risks to users [206]. Recent studies emphasize the need for digitally signed updates, redundant A/B partition storage, and version-controlled rollback to establish a resilient firmware lifecycle [207]. Real-world industry cases demonstrate that when manufacturers discontinue software support or when an update fails, users may be left with "orphaned" or non-maintainable neuro-devices. Therefore, firmware systems for BCIs must simultaneously address security, reversibility, and long-term maintainability.

In non-invasive BCIs, safety failure and protection mechanisms primarily address the risk of unintended operations caused by signal distortion, abnormal user states, or external disturbances. Typical strategies include real-time monitoring of user cognitive states (e.g., fatigue, distraction) and triggering system-level interventions (e.g., halting device operation, switching to manual mode, or activating emergency shutdown) when potentially unsafe conditions are detected. Additionally, multimodal physiological signal fusion (e.g., combining EEG and EOG) or robust control algorithms can enhance system fault tolerance and reduce reliance on a single signal source, allowing basic safety functions to be maintained even when partial component failures occur. Regarding safety thresholds and recovery mechanisms during online learning, systems generally require predefined behavioral boundaries or risk thresholds to constrain algorithmic outputs, preventing learning models or adaptive control strategies from entering hazardous or uncontrollable states. For example, in brain-controlled robots or vehicles, physical or logical limits may be imposed on movement speed, steering angle, or distance to obstacles. When the system detects that its internal state or user commands are approaching or exceeding these limits, recovery procedures are activated, such as aborting current commands, reverting to the last verified safe state, or using error-related neural signals (e.g., error-related negativity) to trigger immediate system reset and behavioral correction. This hybrid strategy—combining proactive threshold protection and reactive error-triggered recovery—ensures that interactions remain within safe operational bounds during dynamic learning [208].

In non-invasive BCIs, online learning effectively addresses the non-stationarity of EEG signals over time, but it introduces key challenges related to safety thresholds and recovery mechanisms. Because EEG signals drift over time, adaptive updates of classifiers are necessary but must avoid performance degradation or user confusion due to improper adaptation. A common approach is to set a confidence threshold: classifier parameters are updated only when the confidence of the current prediction exceeds this threshold, which reduces the likelihood of learning from noisy or uncertain data. Meanwhile, the system must retain recovery capability. For instance, when error-related brain potentials are detected, the system can interrupt current commands and roll back the classifier to a previously validated stable state. This combination of active threshold defense and passive error-triggered rollback aims to balance performance, adaptability, and operational safety [209].

## Conclusions and Perspectives

As non-invasive BCI technology advances from laboratory research toward real-world applications in clinical rehabilitation, neural modulation, and daily life assistance, its evolution underscores a deepening convergence of neuroscience, AI, and flexible bioelectronics. Through systematic advancements in neural decoding algorithms and innovative flexible materials, we are progressively integrating high-precision neural monitoring and decoding technologies seamlessly into daily life, clinical diagnostics, and therapeutic interventions. However, achieving truly sustainable, accessible, and clinically valuable applications still faces a series of challenges rooted in the intrinsic properties of neural signals, the constraints of dynamic human-computer interaction, and the complexity of long-term usage environments. To address these challenges, a systematic and multidimensional breakthrough is required across signal acquisition, algorithmic modeling, hardware architecture, closed-loop control strategies, and evaluation frameworks.

The core performance bottleneck of non-invasive BCIs primarily stems from inherent biophysical constraints. When cortical neural activity passes through multiple tissue layers (e.g., the skull and cerebrospinal fluid), it may cause spatial blurring and amplitude attenuation of the signals. Consequently, scalp-recorded EEG typically exhibits low spatial resolution, low SNR, and high susceptibility to physiological artifacts and environmental noise. Moreover, individual variations in anatomical structure, physiological states (e.g., fatigue, attention), and psychological states (e.g., motivation, emotion) further exacerbate the non-stationarity and time-varying nature of neural signals. Meanwhile, practical challenges at the user level also demand considerable attention. Firstly, there are significant individual differences in neural accessibility. Approximately 15%–30% of users struggle to produce consistently decodable signals, a condition termed "BCI illiteracy." These challenges not only undermine the generalization of decoding algorithms but also hinder the stable and reliable decoding of user intent.

Despite significant advances in neural decoding and system architecture, the large-scale translation of non-invasive BCIs into clinical and everyday settings continues to face substantial challenges. In mobile or real-world usage scenarios, BCI systems confront critical issues related to the stability of the biological interface, which directly impacts long-term reliability and signal quality. Two primary bottlenecks are physical degradation and relative motion at the electrode-scalp interface. Firstly, interface degradation becomes particularly pronounced during prolonged wear. Dry electrodes eliminate the need for conductive gel, thereby reducing the risk of skin irritation or allergic reactions; however, their rigid structures may cause pressure points, skin discomfort, or even micro-abrasions during extended use. Wet electrodes, while offering superior signal fidelity, carry risks of skin sensitization and infection. Moreover, as the conductive gel dries over time, contact impedance increases, leading to progressive signal instability. Semi-dry electrodes represent a compromise solution, slowly releasing minimal electrolytes to maintain conductivity while minimizing skin irritation. However, their design requires careful consideration of material permeability and mechanical configuration to prevent uneven pressure distribution and residue accumulation. To ensure biocompatibility, electrode materials should be selected for low allergenicity, high flexibility, and smooth surface topography. These material properties must be combined with breathable designs, regular cleaning protocols, and personalized fitting procedures to minimize adverse skin reactions and enhance both user comfort and system reliability.

Secondly, dynamic artifacts present another major challenge. Relative movement between the electrode and scalp during user motion not only induces mechanical fatigue and potential structural damage but also introduces motion-related artifacts. Additionally, electrolytes in sweat alter the electrochemical characteristics of the interface, causing drift and interference that further degrade signal quality. Hair acts as a physical barrier, impeding direct contact between the electrode and scalp, increasing contact impedance, and exacerbating artifact magnitude. Collectively, these factors indirectly contribute to feature representation drift and deterioration in decoding performance.

In contrast to conventional rigid electrodes, advancements in flexible bioelectronics are significantly influencing the design principles of neural signal acquisition interfaces. Driven by innovations in materials science, nanomaterials are gaining increasing attention in the development of flexible electrodes. The combination of the unique electrical properties of nanomaterials and the excellent mechanical compliance of flexible substrates provides a solid foundation for high-performance flexible electrodes. The mechanical impedance matching between highly conductive flexible films and biological tissues enables low interfacial impedance and high-fidelity neural signal recording. The conformal contact afforded by such materials helps suppress motion-induced artifacts and maintains more consistent contact impedance. Evidence indicates that this leads to improvements in the SNR and stability of raw EEG signals. Compared to traditional rigid electrodes, flexible electrodes not only enhance signal quality but also offer greater comfort during extended wear, facilitating long-term monitoring.

Nevertheless, even with significant advancements in materials, achieving robust performance across diverse populations still demands fundamental algorithmic breakthroughs. At the preprocessing stage, advanced multimodal fusion algorithms integrate cross-modal sensory information (e.g., visual, auditory, and tactile) to enhance signal robustness and discriminability. During feature extraction, methods based on SPD manifold geometry, CSP and its variants, and spatiotemporal filtering incorporating source imaging priors have improved feature separability and neurophysiological plausibility. In classification, end-to-end deep learning models (e.g., CNNs, Transformer architectures, and multimodal fusion networks) enable high-performance automatic feature learning and pattern recognition, significantly boosting decoding accuracy and generalization. Despite these advances, the efficacy of such models is largely validated under idealized experimental conditions. Their transferability, noise resilience, and long-term stability in real-world deployments remain insufficiently demonstrated.

Concurrently, as BCI technologies develop, ethical, societal, and safety concerns are becoming increasingly prominent. Ultimately, translating advanced laboratory prototypes into clinically validated and commercially viable products requires rigorous scientific validation, comprehensive biocompatibility testing, regulatory approval, and long-term efficacy assessment. Non-invasive BCIs face dual challenges in regulatory clearance and cost modeling for both disposable and reusable components. From an economic perspective, cost models suggest that disposable electrodes increase direct expenditure but may reduce risks of cross-contamination and labor costs associated with cleaning. Reusable systems exhibit lower per-use costs but require investment in sterilization equipment, personnel, and maintenance. An optimized framework should therefore quantify trade-offs among regulatory compliance, clinical safety, and lifecycle cost.

In the development and deployment of non-invasive BCIs, whether as medical devices or consumer-grade products, a systematic checklist encompassing electrical safety, electromagnetic compatibility (EMC), biocompatibility, software lifecycle management, and cybersecurity must be strictly adhered to. For medical devices, patient safety and clinical reliability are paramount, necessitating compliance with IEC 60601 (electrical safety and EMC), ISO 10993 (biocompatibility of electrode patches or wearable materials), IEC 62304 (medical device software lifecycle), and ISO 14971 (risk management). Furthermore, registration with regulatory bodies such as the FDA or under the EU's Medical Device Regulation (MDR) is mandatory, supported by traceable technical documentation and clinical or performance validation data to ensure consistent signal quality, algorithmic stability, and reproducibility across diverse user populations. For consumer-grade products, while full medical device regulations do not apply, essential requirements still hold. These include compliance with electrical safety standards (e.g., IEC 62368 or IEC 61010), EMC and wireless regulations (e.g., FCC/CE RED), and basic safety assessments of skin contact materials (referencing low-risk pathways in ISO 10993). Adherence to data protection laws (e.g., GDPR) and cybersecurity regulations governing data encryption, privacy, and cloud transmission is equally critical. Additionally, commercial products must avoid using diagnostic or therapeutic terminology, instead positioning their functionality as cognitive state monitoring, wellness support, or entertainment, to mitigate the risk of being classified as medical devices. Moreover, non-invasive BCIs must adhere to strict disinfection and sterilization protocols in clinical and research settings to ensure participant safety, health, and ethical protection.

Particularly crucial is that current BCI research is undergoing a fundamental shift from "open-loop, static" systems to "closed-loop, adaptive" paradigms, with a focus on enhancing real-time decoding capabilities, improving asynchronous detection mechanisms, and optimizing shared control strategies. However, a key engineering constraint persists: phase lag. This lag, which includes the cumulative time delay arising from signal acquisition, wireless transmission, feature extraction, decoding decisions, and actuator response, causes the system output to trail behind the user's neural intention. This temporal misalignment disrupts control timing, reduces trajectory smoothness and target acquisition accuracy, and significantly diminishes the user's sense of agency, thereby undermining trust and sustained engagement. In high-speed motor control or high-risk tasks, even minor delays can lead to serious consequences. Moreover, an inherent trade-off exists between latency and reliability: complex models improve classification accuracy but often incur higher computational overhead and longer response times, whereas simplified algorithms reduce latency at the expense of decoding robustness. Of equal concern is the nonlinear accumulation of false positives (false triggers) and false negatives (missed detections) during extended use, which increases cognitive load and may even lead to user frustration or abandonment. Meanwhile, the balance of control authority in shared control paradigms remains lacking in standardized definitions. To address these challenges, forward models and predictive state filters should be incorporated to model user intent, generate preemptive control commands, actively compensate for cumulative processing delays, and thereby enhance the proactivity and responsiveness of closed-loop systems. Using preregistration or at minimum a constrained analysis plan will help limit post hoc selection.

Looking ahead, open research benchmarks should be established to promote reproducible, comparable, and verifiable progress. Pre-registered or constrained analysis plans are recommended to limit post hoc selection bias. In addition, the biological interpretability of BCI systems should be rigorously assessed by evaluating the stability of channel or regional weights across repeated sessions and across different subjects. Furthermore, comprehensive, endpoint-aligned evaluation systems should be developed, integrating technical metrics with task completion time, false alarm rates, subjective measures such as the NASA-TLX cognitive load scale, and long-term usage rates, thereby establishing user-centered evaluation criteria.

At the system architecture level, advancing beyond current performance limitations requires the development of specialized hardware designed for emerging computing paradigms. The traditional von Neumann architecture faces energy efficiency bottlenecks when processing neural signals, whereas neuromorphic computing approaches, such as those utilizing memristor-based in-memory computing chips combined with event-driven spiking neural networks, provide a promising solution for ultra-low-power edge computing. This necessitates hardware-software co-design, involving cross-level optimization from materials and devices to algorithms. Such co-design ensures integrated consideration of hardware physical characteristics and algorithmic computational requirements from the initial design stages, while minimizing latency and privacy risks associated with cloud transmission.

In summary, the advancement of non-invasive BCIs relies not only on innovations in signal processing and algorithm design but also on close integration with cutting-edge flexible bioelectronics and hardware architecture. Achieving practical, robust, personalized, and trustworthy BCI systems capable of transitioning from the lab to real-world deployment requires multidisciplinary collaboration and holistic co-optimization. This process involves not only technological innovation but also strict regulatory compliance, cost-benefit analysis, and user experience improvement to ensure safety, reliability, and widespread accessibility. Simultaneously, establishing comprehensive end-to-end evaluation systems that span from signal acquisition to final user feedback is essential for validating the effectiveness and reliability of diverse hardware-software configurations and will serve as a key focus for the future development of BCI technologies.
